# Thorough Validation of Optimized Size Exclusion Chromatography-Total Organic Carbon Analysis for Natural Organic Matter in Fresh Waters

**DOI:** 10.3390/molecules29092075

**Published:** 2024-04-30

**Authors:** Elien Laforce, Karlien Dejaeger, Marjolein Vanoppen, Emile Cornelissen, Jeriffa De Clercq, Pieter Vermeir

**Affiliations:** 1 Industrial Catalysis and Adsorption Technology (INCAT), Department of Materials, Textiles and Chemical Engineering, Faculty of Engineering and Architecture, Ghent University, 9000 Ghent, Belgium; 2 PaInT, Particle and Interfacial Technology Group, Department of Green Chemistry and Technology, Faculty of Bioscience Engineering, Ghent University, Coupure Links 653, 9000 Ghent, Belgium; 3 Centre for Advanced Process Technology for Urban Resource Recovery (CAPTURE), Frieda Saeysstraat 1, 9052 Ghent, Belgium; 4 CNRS, UMR 8516—LASIRE—Laboratoire Avancé de Spectroscopie pour les Intéractions la Réactivité et l’Environnement, Université de Lille, 59000 Lille, France; 5 KWR Water Research Institute, Groningenhaven 7, 3433 PE Nieuwegein, The Netherlands; 6 Laboratory for Chemical Analysis (LCA), Department of Green Chemistry and Technology, Faculty of Bioscience Engineering, Ghent University, 9000 Ghent, Belgium

**Keywords:** chromatographic fractionation, method validation, precision, trueness, recovery, sample preservation

## Abstract

Size exclusion chromatography with total organic carbon detection (HPSEC-TOC) is a widely employed technique for characterizing aquatic natural organic matter (NOM) into high, medium, and low molecular weight fractions. This study validates the suitability of HPSEC-TOC for a simplified yet efficient routine analysis of freshwater and its application within drinking water treatment plants. The investigation highlights key procedural considerations for optimal results and shows the importance of sample preservation by refrigeration with a maximum storage duration of two weeks. Prior to analysis, the removal of inorganic carbon is essential, which is achieved without altering the NOM composition through sample acidification to pH 6 and subsequent N_2_-purging. The chromatographic separation employs a preparative TSK HW-50S column to achieve a limit of detection of 19.0 µgC dm^−3^ with an injection volume of 1350 mm^−3^. The method demonstrates linearity up to 10,000 µgC dm^−3^. Precision, trueness and recovery assessments are conducted using certified reference materials, model compounds, and real water samples. The relative measurement uncertainty in routine analysis ranges from 3.22% to 5.17%, while the measurement uncertainty on the bias is 8.73%. Overall, the HPSEC-TOC represents a reliable tool for NOM fractions analysis in both treated and untreated ground and surface water.

## 1. Introduction

Natural organic matter (NOM) is a very complex mixture of aromatic and aliphatic compounds ranging from a few hundreds to 100,000 Da in molecular weight (MW) [1]. It is present in all natural water sources causing water quality issues such as taste and odour problems, bacterial regrowth or disinfection by-product formation in drinking water [2,3]. Currently, routine analysis in drinking water treatment plants focus on the assessment of bulk total organic carbon (TOC) or ultraviolet (UV) absorption (usually 254 nm) [4]. However, these parameters often fall short in predicting the influence of NOM on the water treatment efficiency and/or on potential water quality issues, since these processes and/or issues are usually linked to specific NOM fractions [3,5]. As such, recent research described the correlation between specific NOM fractions and the formation of disinfection by-products [3]. Consequently, fluctuations in both NOM concentration and characteristics caused by e.g., seasonal variations introduce additional challenges in drinking water production to regulate the formation of disinfection by-products and/or other water quality parameters. Moreover, climate change will cause an increase in NOM concentration and alterations in NOM composition in the drinking water sources [4,6]. These issues clearly demand for a more detailed NOM monitoring.

Many characterization methods are available to describe the behaviour and fate of NOM in natural waters. Advances in pyrolysis-gas chromatography-mass spectrometry (Py-GC-MS), fluorescence spectroscopy, nuclear magnetic resonance (NMR) spectroscopy, high-resolution Fourier-transform ion cyclotron resonance mass spectrometry (FTICR-MS) and high performance size exclusion chromatography (HPSEC) methods have been explored to provide unique insights into NOM characteristics [3,7]. Significant temporal and spatial variations in NOM composition have been unravelled using a combination of these techniques providing high analytical resolution [3]. Moreover, molecular formulae of a universal NOM component present in both oceans, rivers and lakes have been identified through FTICR-MS and NMR technology [8]. Most of these techniques, such as py-GC-MS, NMR and FTICR-MS are, however, not appropriate for routine monitoring due to their cost, complexity, analysis time and sophisticated analytical instrumentation [2,3]. Furthermore, some of these methods such as FTICR-MS require desalting and concentration of the sample with solid phase extraction, which results in partial NOM extraction [3,7].

The remaining characterization method, i.e., HPSEC, is a suitable candidate for routine, long-term monitoring of NOM in water treatment processes and has been proven useful to assess removal efficiencies in typical drinking water treatment plants [9,10,11]. Its efficacy lies in its capability to separate NOM in different fractions based on molecular size and shape [7]. A wide range of detectors can be connected to this system such as photodiode arrays, mass spectrometry or fluorescence detection [7,12,13,14,15,16,17], however, the most commonly ones include UV and organic carbon detectors (OCDs). Although UV detectors are simple and fast, they are limited to detect molecules that absorb light at a specific wavelength (usually 254 nm). OCDs provide better quantitative information with a low detection limit [7,12,13,14,15,16,17]. Nevertheless, the typically low concentration of NOM in natural waters introduces analytical challenges. Conventionally used concentration methods such as solid phase extraction may introduce alterations to the complex NOM mixture [18,19]. Hence, it is preferable to avoid concentration steps and instead opt for a larger injecting volume of the original sample and a (semi-) preparative column for HPSEC analysis [20].

Liquid chromatography (i.e., HPSEC) coupled to an organic carbon, organic nitrogen (OND) and UV (254 nm) detector (LC-OCD-OND) is the most widely used method to characterize NOM in fresh water samples. The LC-OCD-OND method divides NOM into six fractions [21]. The first fraction is referred to as hydrophobic NOM, which is not detected during the timespan of the analysis because of its relatively strong interactions with the stationary phase. The other five fractions found in the OCD chromatogram, in order of elution from the column, are biopolymers, humic substances, building blocks, low molecular weight (MW) acids and neutrals. The separation between humic substances, building blocks and low MW acids is done through deconvolution of the OCD chromatogram and UV absorbance at 254 nm, where the humic substances peak is the most apparent having two shoulders for the building blocks and low MW acids respectively [21]. Humic substances mainly consist of humic and fulvic acids and are therefore more hydrophobic structures containing phenolic and carboxylic functionalities, of which the latter are anionic under neutral pH conditions. Building blocks are considered as degradation products of humic substances, thus having lower MW but similar characteristics. Low MW acids are defined as relatively small molecules which are negatively charged at neutral pH. Due to their anionic properties, they are slightly repulsed by the packing material of the SEC column under the used conditions [22], resulting in shorter elution times than expected based on their MW. This has been proven by Ruhl et al. (2012), who investigated the elution behavior of a large set of low MW acid compounds with LC-OCD-OND and showed that their elution occurs over the entire humic substances peak and not only in the shoulder, which is denoted as the low MW acid peak [23,24]. LC-OCD-OND has been applied in various studies to investigate the performance of drinking water treatment processes and/or to assess the impact of altering NOM characteristics in the source water on these processes [25,26,27,28], e.g., MacKeown et al. (2021) found a correlation between trihalomethane formation potential and the concentration of building blocks in the water [26]. A drawback of LC-OCD-OND is that its implementation and data interpretation are still relatively complex, which impedes its use in routine analysis. This complexity arises from the use of deconvolution and the combination of three different detection methods.

When a new HPSEC method is presented or applied in scientific publications, an extensive validation is very often lacking [2,12]. Nevertheless, validation is of key importance when applying a new method for routine analysis to ensure the quality, improve process optimization and to provide good science [29]. Validation should be conducted when using non-standard methods or after modification of standard methods and results in a level of confidence because it shows that the performance characteristics are fit for use for a particular measurement [30]. Common validation parameters that should be determined when implementing a new method are the trueness, precision, linearity, limit of detection (LOD), limit of quantification (LOQ) and robustness [29]. Trueness is measured by defining the percent recovery of the analyte at different concentration levels that are covered in the analytical method. Precision includes repeatability, intermediate precision and reproducibility and is expressed as variance, standard deviation or coefficient of variation over a series of samples. With robustness, it is verified if the analytical method stays unaffected when small variations in the method parameters occur such as the stability of analytical solutions [29]. (Fresh) water samples are very susceptible to changes which might take place between sampling and measurement [31] or by freezing and thawing the sample [32]. Therefore, assessing the stability of a sample is also a key practice when validating a method that analyzes fresh water samples [31,32]. Together, all these validation parameters indicate the performance of an analytical method for a specific type of sample. Nonetheless, HPSEC-TOC systems have only been validated in terms of LOD and repeatability by Dulaquais et al. (2018), who developed a slightly modified LC-OCD-OND system for the analysis of estuarine and marine water [33]. For fresh water analysis, available validation parameters for LC-OCD-OND are limited to repeatability data [21]. Hence, an extensive validation of HPSEC-TOC systems for fresh water samples has not yet been published to the best of our knowledge.

Therefore, this work proposes an HPSEC-TOC method for routine measurements of NOM fractions in fresh waters. The system is connected to only one detector which quantifies all organics, in contrast to UV detection which only measures UV active compounds. The chosen TOC detector uses UV/persulfate oxidation, i.e., it only needs an acid and oxidant and no carrier gas supply, which makes it compact, simple and easier to implement. A preparative column is selected based on the set-up and sensitivity of the detection system and to avoid the necessity of pre-concentration of the sample. This simplified set-up lowers the maintenance costs and facilitates straightforward and quick data processing, all key advantages for routine analysis. The method is developed and validated in terms of sample pretreatment (removal of inorganic carbon (IC)), trueness, precision, LOD, LOQ, method uncertainty and sample stability by investigating different storage methods and storage times.

## 2. Results and Discussion

### 2.1. Method Development

#### 2.1.1. HPSEC-TOC Calibration

The HPSEC-TOC method was calibrated on an organic carbon basis with potassium hydrogen phthalate (KHP). The measurements based on concentration showed linearity of the calibration in a concentration range of 0.05 to 10.00 mgC dm^−3^ (R^2^ = 0.999) for a single molecule peak (see Figure S1) satisfying the >0.998 criterion for adequate linearity [34]. For the MW calibration, two calibrants were compared to each other. Polyethylene glycol (PEG) is one of the typically used calibrants for HPSEC-TOC systems [2,15,20,35], while pullulan has not been reported to the best of our knowledge. Their chemical structures can be found in Figure S2. Both compounds are hydrophilic, but pullulan, a polysaccharide, is structurally more similar to the biopolymer or high MW fraction, which mostly consists of polysaccharides and some proteins [36]. PEG is a linear alcohol and can hence be considered as a surrogate for the hydrophilic low MW fraction.

Polystyrene sulfonate, a hydrophobic but water soluble polymer, has been widely used as calibrant [16,20,37,38,39,40] since it is UV-active and its structure is similar to hydrophobic humic substances, which are the most abundant fraction in a NOM mixture (Figure S2) [36]. However, sulfonic acid has a much lower pK_a_ value compared to the acid functionalities present in humic substances such as carboxylic acid and phenolic groups [41] and will therefore have a high repulsion with the stationary phase, resulting in a faster elution [15,20]. Hence, it is expected that the elution behaviour of humic substances will match better with the PEG or pullulan MW calibrants than with the polystyrene sulfonate.

**Figure 1:**
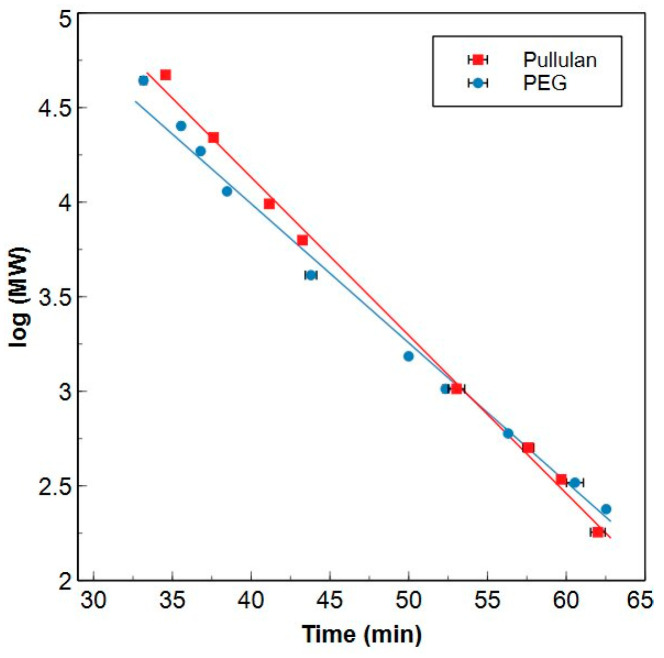
The apparent molecular weight (MW (Da)) versus elution time for pullulan (red squares, y = −0.084x + 7.503, R^2^ = 0.997) and PEG standards (blue dots, y = −0.076x + 7.041, R^2^ = 0.992). Average elution times of two independent analyses are presented together with the respective standard deviations.

The calibration curves for PEG and pullulan are presented in Figure 1. Figure S3 shows a chromatogram of a water sample where the TOC intensity is plotted against the elution time and the apparent MW according to the PEG and pullulan calibrations. A similar elution behaviour (deviation of calculated elution times < 10%) is observed for both calibrants for moderate MWs (≈0.300–2.00 kDa). This in in accordance with the findings reported by Agilent concerning the elution behaviour of these two calibrants when silica particles coated with a hydrophilic layer are used as stationary phase [42]. For MWs that exceed the upper limit of this interval (>2.00 kDa), it can be noted that a pullulan standard will elute later compared to a PEG standard with a similar MW. For the lower MWs (<0.300 kDa), PEG standards will elute later compared to pullulan. This indicates that, especially for the relatively high or low MWs, the calibrant needs to be selected based on the fraction of interest. Since the chemical structure of pullulan is most similar to the high MW biopolymer fraction, pullulan is preferred when targeting compounds with higher MWs. However, PEG results in a more reliable apparent (a)MW for smaller molecules having similar structures as this calibrant.

The calibration curves can be used for the determination of the (a)MW of a molecule or the main (a)MW and MW distribution of a mixture. Only MW-ranges are determined in this work due to the complexity of NOM mixtures. (a)MWs are linked to the elution times and integration ranges mentioned in this work to enable the interlaboratory comparability of the used HPSEC-TOC system. Elution times are system-specific since it depends on both the separation itself as well as on constant but system-related parameters such as the length of the tubing between the SEC column and the detectors. (a)MWs are not influenced by these parameters and are hence more relevant when aiming at interlaboratory comparison or validation. Since the two calibrants show high linearity, it was decided to determine the (a)MW range for the high MW fraction with pullulan, while the (a)MW range for the low MW fraction will be based on the PEG calibration curve (Figure 1). It is advised to perform a MW calibration on regular time-intervals as a quality control for the separation performance of the SEC-column.

#### 2.1.2. Definition of the Integration Ranges

**Figure 2:**
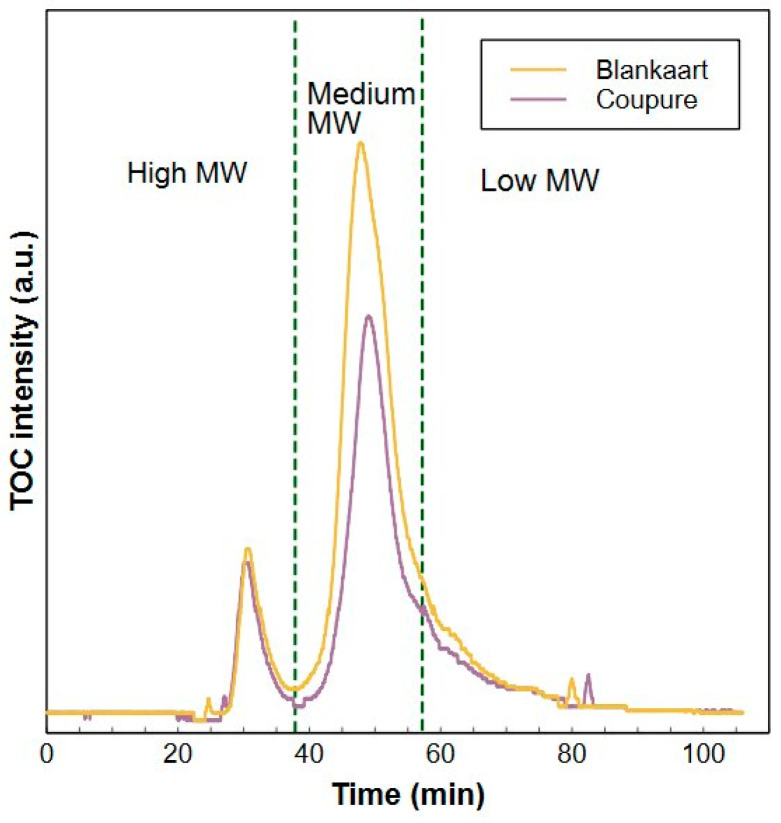
Visualisation of the three integration zones for Blankaart (orange) and Coupure (purple) water.

The main advantage of a (SEC) separation of organic matter in a water sample before conducting the TOC analysis is the additional information about the characteristics or composition that can be acquired next to the quantitative information. Defining the number of fractions, and hence integration ranges in the SEC chromatogram, is important since more fractions will result in a more complex data set, complicating the interpretation and increasing the labor time, especially for routine analysis. Therefore, it was decided to differentiate three NOM fractions with similar characteristics in this method (as indicated in Figure 2) instead of the five fractions typically reported in LC-OCD-OND [21].

The Sievers^®^ M9 TOC detector was chosen as online detection system to provide an accessible and easy to implement tool for HPSEC-TOC analysis. The TOC detector used in this work is more compact compared to the OCD detector. Moreover, no carrier gasses are required for its operation, while a N_2_-gas flow is used in the OCD system. These factors facilitates its fast, safe and economical beneficial implementation in a laboratory.

- High MW fraction

Molecules with hydrodynamic volumes that exceed the exclusion limit of the SEC column (and which do not strongly interact with the stationary phase) elute together in one peak, ending at 38.0 min in the HPSEC-TOC analysis. This integration range matches with the biopolymer fraction in LC-OCD-OND analysis which uses the same SEC column and similar operating conditions [21]. This range corresponds with molecules having an (a)MW > 20.3 kDa with the pullulan calibration and consists mainly of proteins and polysaccharides [36].

b. Medium MW fraction

This integration range (38–59.5 min) includes humic substances, building blocks and low MW acid fractions as reported in LC-OCD-OND [21]. Distinction between those fractions is intentionally not made in this HPSEC-TOC system. The conductivity of the sample is adjusted to the one of the mobile phase to suppress matrix effects which assures an accurate detection, but results in a decreased resolution of these three fractions. Merging the three fractions to one combined fraction is however justified by the following reasons; Firstly, the three fractions have similar chemical structures (e.g., aromatic character of humic substances and building blocks) and/or functionalities (e.g., presence of carboxylic acid functionalities). This implies a similar behavior during different water treatment processes such as their removal via enhanced coagulation [43]. Moreover, the elution time of the low MW acids can vary largely due to their different charge densities and pKa values [23]. Lastly, the relatively low concentration of low MW acids in fresh water samples results in a very low repeatability of this fraction in the LC-OCD-OND system (relative standard deviation (RSD) = 19.8%, see Section 2.2.2.c). Overall, combining the three fractions results in more straightforward, reproduceable and simplified analysis. It can be noted that applications where the main targeted compounds are specifically building block or low MW anionic compounds in the low ppb-level can still benefit from the LC-OCD-OND analysis. Molecules eluting between 38 min ((a)MW < 20.3 or 14.6 kDa with pullulan or PEG calibration respectively) and 59.5 min ((a)MW > 0.316 or 0.286 kDa with pullulan or PEG calibration respectively) are classified as the medium MW fraction in the HPSEC-TOC method. The lower limit of this interval was defined based on the elution time of salts such as (sodium) bicarbonate on the one hand and low MW monovalent acids such as fumaric acid on the other hand (Figure S4).

c. Low MW fraction

All molecules eluting later than 59.5 min ((a)MW < 0.286 kDa with PEG calibration) are categorized as the low MW fraction. This integration range (>59.5 min) is similar to the low MW neutral fraction in LC-OCD-OND [21]. Molecules eluting in this range have a small hydrodynamic volume and are uncharged at neutral pH or are relatively hydrophobic. The latter causes a certain retention by the stationary phase resulting in later elution times than expected based on the MW [21,23]. Even though a buffer solution is used as mobile phase and sample matrices are aligned with the mobile phase of the system, interactions with the SEC resin used as the stationary phase are not completely suppressed.

#### 2.1.3. HPSEC-TOC Sample Pretreatment

**Figure 3:**
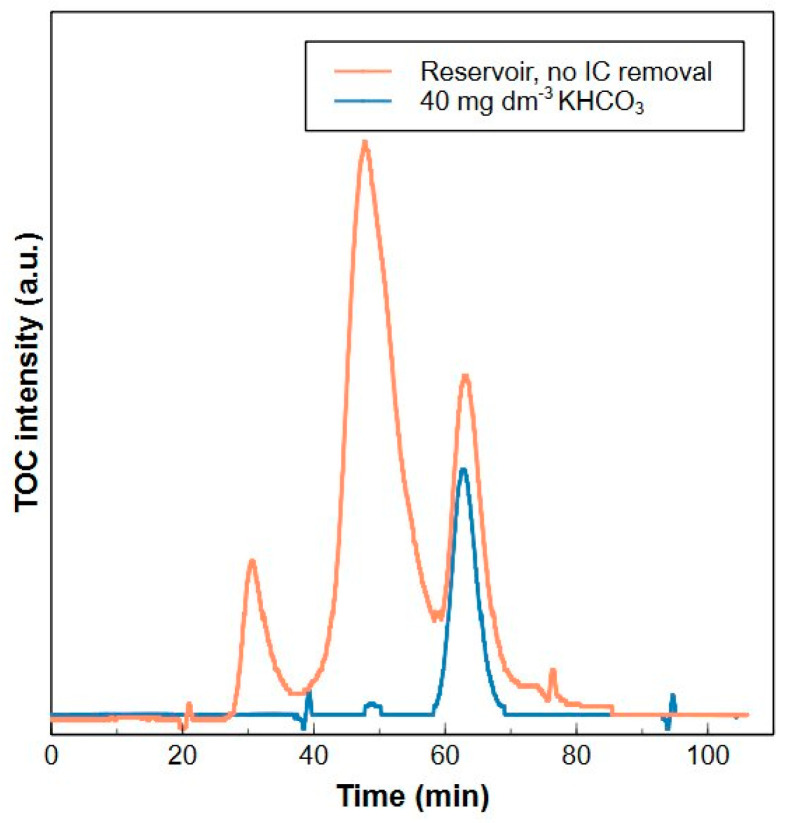
HPSEC-TOC chromatograms from Blankaart reservoir water (orange) and from a 40 mgC dm^−3^ KHCO_3_ solution (blue). The peak at 68 min is assigned to the incomplete removal of inorganic carbon.

During a HPSEC-TOC analysis, the organic carbon concentration is measured every 4 s and calculated from the difference between total carbon and total IC [44]. A high concentration of IC will therefore affect the organic carbon measurement. An IC remover is installed inside the Sievers^®^ M9 detector to remove the IC prior to TOC measurement. A degassing unit converts IC into CO_2_ by adding acid to the water which is subsequently removed by vacuum treatment. However, it was observed that the tube degassing unit of the Sievers^®^ M9 detector could not sufficiently remove the IC from the samples within the operating conditions (flowrate = 1 cm^3^ min^−1^). In Figure 3, a large peak around 68 min is seen in the HPSEC-TOC chromatogram of a 40 mgC dm^−3^ KHCO_3_ solution, which clearly shows the incomplete removal of IC. The peak is also observed in a Blankaart water sample which contained about 50 mgC dm^−3^ of IC (Figure 3, Table S1).

Therefore, a standard series of different IC concentrations (3–85 mgC dm^−3^) in milliQ were analyzed with the HPSEC-TOC to determine the maximum amount of IC that can be removed by the degassing unit (Figure S5). The removal efficiency was on average 94.6% with a maximum of 97% for the lowest concentration (3 mgC dm^−3^). If the removal is not 100%, an interfering peak is detected in the low MW fraction, as shown in Figure 3. This was also observed in literature [15].

Therefore, acidification coupled to nitrogen purging or sonication in vacuum was investigated to remove IC from fresh water samples prior to analysis. H_3_PO_4_ was chosen to acidify the samples, because it is compatible with the mobile phase and the organic carbon detection system of the HPSEC method. Furthermore, the acidification was limited to pH 5 to avoid possible changes in NOM composition. It is known that e.g., humic acids (part of the humic substances, eluting in the medium MW fraction) precipitate at pH 2 or lower [36,45]. Moreover, a pH below the pKa of a molecule results in complete protonation. Carboxylic acids are the most abundant functionalities in humic substances and have a pKa around 4.7. A pH modification to pH 2.5 of a water sample therefore results in excessive protonation of the humic substances. The buffering capacity of the concentrated buffer used to pretreat the sample before analysis is not sufficient to overcome such a low pH and consequently, a shift in elution time of the medium MW fraction is observed in Blankaart water at pH 2.5 (Figure S6). This is also previously observed by Cai et al. (2020) who used a pH of 3 to remove the IC prior to analysis, which caused a change in the profile of the building blocks [15].

**Figure 4:**
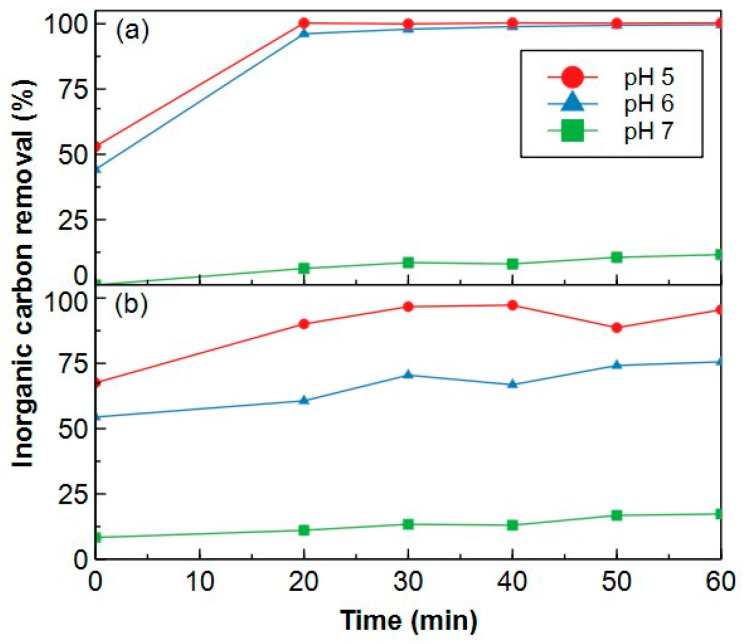
Removal of inorganic carbon from Coupure water (contained ± 60 mgC dm^−3^ of inorganic carbon) in time during (**a**) nitrogen purging and (**b**) sonication in vacuum. The sample was acidified with 0.1 M H_3_PO_4_ to pH 5 (●), pH 6 (▲) and pH 7 (■).

**Figure 5:**
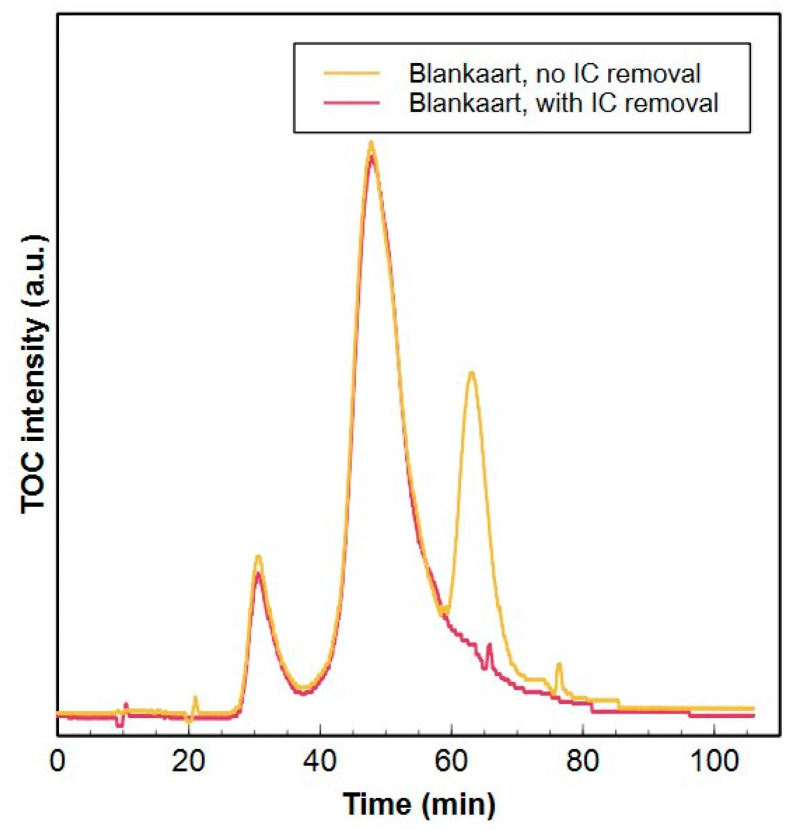
HPSEC-TOC chromatograms from Blankaart water with (red) and without (yellow) inorganic carbon removal prior to analysis. Inorganic carbon (IC) was removed, as observed by the disappearance of this particular peak at 68 min. No other changes in natural organic matter composition occurred after acidification and nitrogen purge.

Figure 4 shows the removal of IC for nitrogen purging and sonication in vacuum treatment in function of pH for Coupure water with an initial IC concentration of ±55 mgC dm^−3^ (Table S2). Neither nitrogen purge nor sonication in vacuum could remove considerable amounts of IC at pH 7. On the other hand, water samples acidified to pH 5 and 6 contained less than 1 mgC dm^−3^ (98–100% removed) of IC after 30 min of nitrogen purge (Figure 4a), while sonication in vacuum only removed between 55–75% of IC at pH 6 and between 67–97% at pH 5 over the 60 min of operation (Figure 4b). Equilibrium between H_2_CO_3_ and HCO_3_^−^ is reached around pH 6.5 [46]. Therefore, at pH 5 and 6, IC will be mostly present as H_2_CO_3_, promoting the conversion towards H_2_O and CO_2_. Moreover, nitrogen purging clearly acts as a better driving force towards gaseous CO_2_ compared to sonication with vacuum. Based on these results, it was decided to remove the IC from the samples by acidification to pH 6 coupled to nitrogen purging for 30 min. This procedure was repeated with Blankaart water. The sample was acidified to a pH of 6, purged with nitrogen for 30 min and subsequently analysed with HPSEC-TOC to verify if (i) all IC was removed and (ii) no changes in NOM composition occurred during this pretreatment. The chromatograms of the untreated surface water and the acidified sample are shown in Figure 5 where the disappearance of the inorganic peak at 68 min is clearly observed without any changes in terms of NOM composition. The pretreatment with nitrogen at pH 6 will therefore be used in further analysis.

### 2.2. Method Validation

#### 2.2.1. LOD and LOQ Determination

The LOD and LOQ of the HPSEC-TOC method calculated from the data of the 250 µgC dm^−3^ KHP standard are respectively 19.0 µgC dm^−3^ and 63.2 µgC dm^−3^ which are adequate values for the analysis of raw and treated surface and ground water samples. These values are higher than the LC-OCD-OND system where concentrations in the low µgC dm^−3^ range are reported [33]. The reason for the lower LOD and LOQ in the LC-OCD-OND system is due to the different TOC detector system used (see Section 2.2.2c).

#### 2.2.2. Precision and Trueness

- Certified reference material

**Figure 6:**
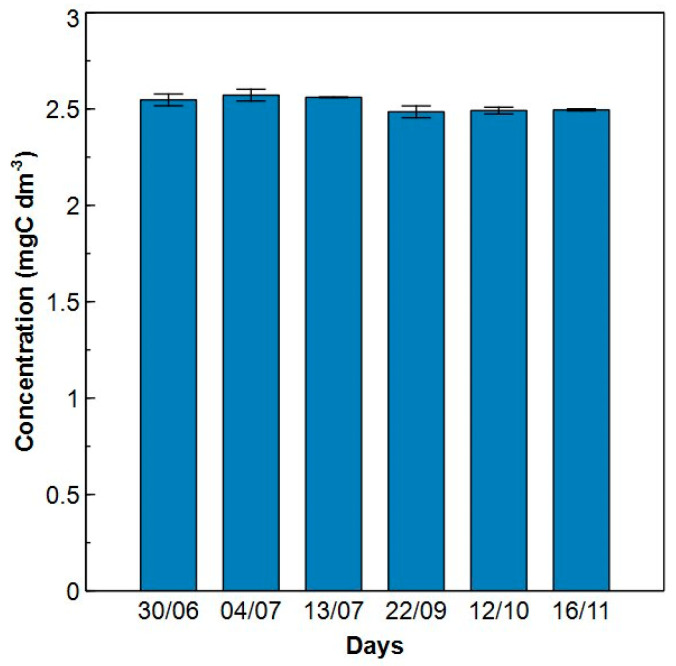
Repeatability of the HPSEC-TOC method using a 2.50 mgC dm^−3^ potassium hydrogen phthalate standard solution over a time span of 20 weeks in 2022. No statistical difference was observed over the different weeks. The relative standard deviation over all measurements was 1.61% and the recovery was 101 ± 2%. Error bars show the standard deviation from duplicate analysis of independent samples.

The repeatability of the HPSEC-TOC method was first verified using a certified reference material (KHP). A concentration of 2.50 mgC dm^−3^ was measured in duplicate on six different days over a period of 20 weeks (Figure 6). No statistical difference was found between the six different measurements. The RSD (standard deviationmean×100%) over all measurements was 1.61% and the recovery of KHP was 101 ± 2%. This is within the acceptance criterium (RSD < 15%), meaning that the system is precise and accurate in measuring KHP concentrations over a period of at least 20 weeks [32].

b. Model compounds

**Figure 7:**
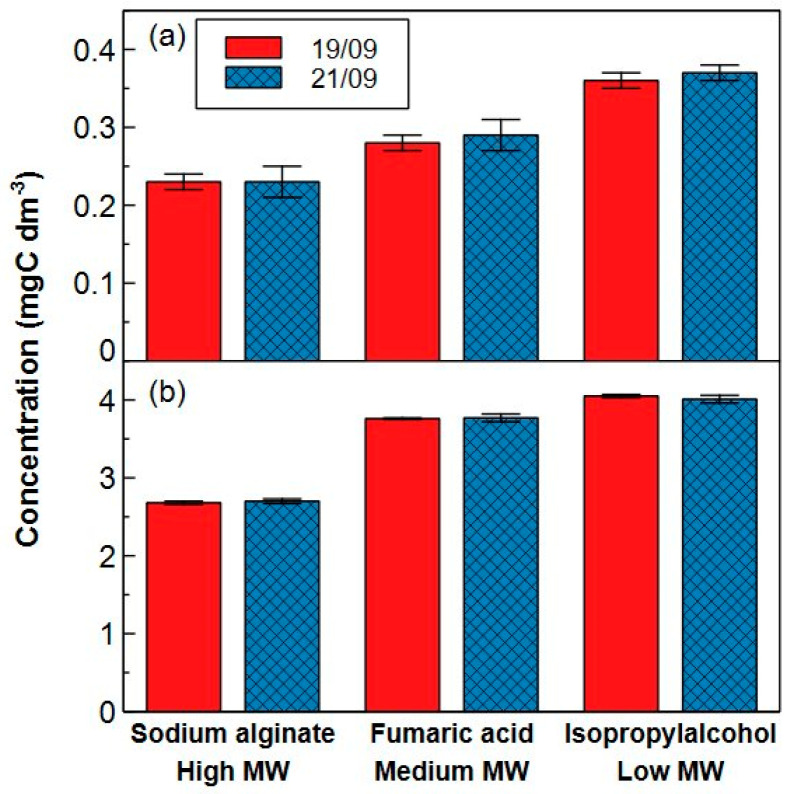
(Intra-)repeatability test for the HPSEC-TOC method with molecules eluting in the high (sodium alginate), medium (fumaric acid) and low (isopropyl alcohol) molecular weight (MW) ranges at a concentration of (**a**) 0.4 mgC dm^−3^ and (**b**) 4.0 mgC dm^−3^ measured on two different days in 2022 (19/09 = solid, 21/09 = crossed bars). No statistical differences were observed between the days. Error bars show the standard deviation from triplicate analyses of independent samples.

Next to the certified reference material, the repeatability of the analytical method was determined by measuring 3 model compounds (sodium alginate, fumaric acid and isopropyl alcohol), eluting in respectively the high, medium and low MW fraction, at 0.4 and 4.0 mgC dm^−3^. The RSDs at 0.4 mgC dm^−3^ were 3.73%, 4.39% and 1.53% for sodium alginate, fumaric acid and isopropyl alcohol respectively. The RSDs at 4.0 mgC dm^−3^ were 0.56%, 0.26% and 0.51% (19/09 on Figure 7). All RSDs are <15% and within the acceptance criterium for precision [32].

The intra-repeatability was determined by measuring the same concentrations of the three model compounds two days later (21/09 on Figure 7). No significant differences could be found (*p* > 0.05) between measurements executed on different days, confirming the reliability of the method.

**Figure 8:**
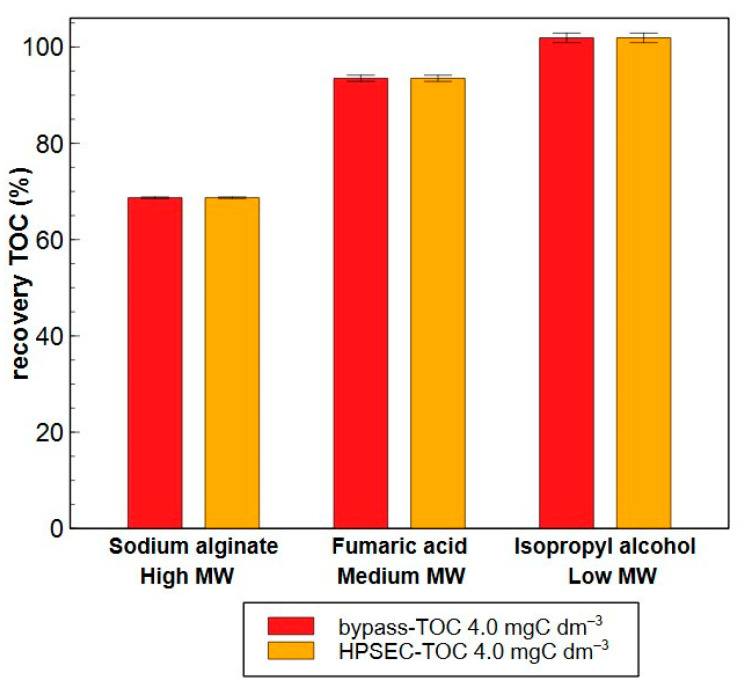
Recovery of 4.0 mgC dm^−3^ solutions of sodium alginate, fumaric acid and isopropyl alcohol determined during HPSEC-TOC with the column bypassed (red) and normal HPSEC-TOC mode (orange).

However, Figure 7a shows that for all compounds, the measured concentration is lower than the targeted concentration (0.40 mgC dm^−3^), while for higher concentrations (4.0 mgC dm^−3^), this is specifically the case for sodium alginate. The exact recoveries of each compound are presented in Table 1. Incomplete recoveries might be caused by insufficient oxidation, but also by the interactions of a molecule with the stationary phase. It is expected that a low recovery of a hydrophobic molecule is contributed by strong interactions with the stationary phase while a poor recovery of a hydrophilic, but larger and more complex molecule is mainly allocated to a low oxidation yield. In this regard, larger molecules such as tannic acid have already been reported to have lower oxidation yields during UV/persulfate oxidation (or wet-chemical oxidation), while small molecules such as glucose, phenylalanine or isopropyl alcohol reach oxidation efficiencies between 90–100% [47,48]. The observed recoveries in this work are in line with the findings of Li et al. (2019), who investigated the recovery of 14 nitrogen-containing organic model compounds such as humic acid, proteins, polysaccharides and amino acids with LC-OCD-OND [46]. These authors found that the majority of these compounds exhibited recoveries between approximately 70 and 105%. However, compounds with higher MWs deviated from this range, achieving recoveries of no more than 25–50%. This was primarily attributed to the lower UV oxidation efficiency in the Gräntzel reactor of the LC-OCD-OND system for these complex, high molecular weight compounds [49]. To determine if the incomplete recoveries are due to adsorption or oxidation, a new 4.0 mgC dm^−3^ solution of sodium alginate, fumaric acid and isopropyl alcohol was first measured in the HPSEC-TOC set-up where the column is by-passed, and then compared with a normal HPSEC-TOC measurement to assess the fraction that is adsorbed on the column (Figure 8). The recovery of sodium alginate in by-pass reached 75% instead of 65% in normal operation, meaning that the main cause of the incomplete recovery (25%) is a low oxidation yield, while interactions with the stationary phase are rather small (10% recovery loss). Although a recovery close to 100% is preferred, it was still higher than 50%, complying with reported guidelines [32]. No difference in organic matter concentration is observed for fumaric acid and isopropyl alcohol suggesting that these compounds are completely oxidized in the detector and possible losses in recovery are due to interactions with the stationary phase. Figure S7 shows the absolute losses in recovery (expressed as mgC dm^−3^) for the different compounds. Here, the absolute loss of sodium alginate increases almost linearly with concentration (≈8× more compound loss with a 10× increase in concentration), while the absolute losses of fumaric acid only doubles with a 10-fold increase in concentration. This implies that for fumaric acid, a small share of this compound is lost by interaction with the column, resulting in a higher portion of recovery loss (expressed in %) at lower concentrations.

c. Real water samples

A final check for the repeatability was done by analysing a Blankaart water sample six times. The RSDs were 4.63%, 1.59% and 5.42% for the high, medium and low MW fraction. The RSD of the total DOC was 1.53%, confirming the precision of the method for real water samples (RSD < 15%) [32]. Huber et al. (2011) also examined the repeatability of the LC-OCD-OND technique with real water samples and reported RSDs of 1.5%, 3.5%, 1.7%, 4.9%, 19.5% and 7.3% for the total DOC, biopolymers, humic substances, building blocks, low MW acids and neutrals [21], while Dulaquais et al. (2018) reported RSDs for seawater of 3%, 9%, 2%, 8%, 5% for total DOC, biopolymers, humic substances, building blocks and low MW neutrals. The relatively high deviations for LC-OCD-OND for especially the low MW acid fraction in fresh waters, which is close to the acceptance level of 20% near LOQ, illustrate that making a reproducible and reliable differentiation between five fractions is challenging [32].

**Figure 9:**
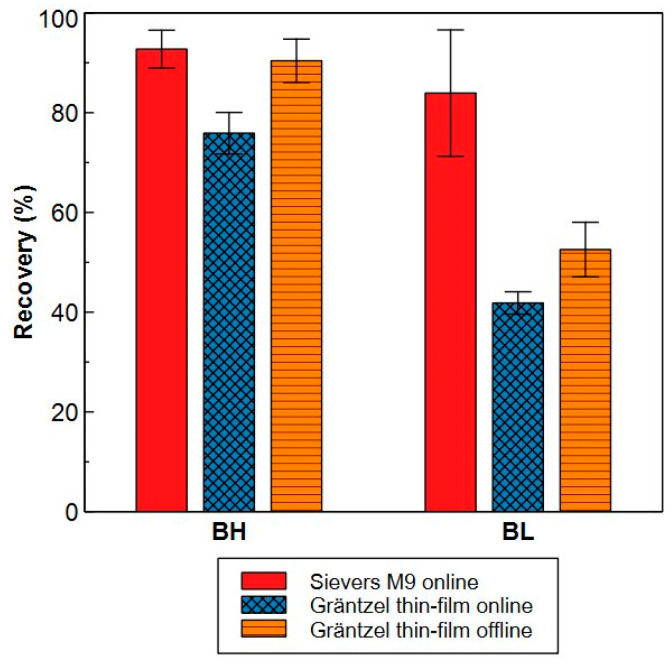
Recovery of different TOC detectors and/or systems compared to the Sievers^®^ 900 offline detector as a reference of 100% for chemical oxidation. Solid bar = Sievers^®^ M9 detector in online mode. Crossed bar = Gräntzel thin-film reactor in online mode. Lined bar = Gräntzel thin-film reactor in offline mode. BH= similar Blankaart samples with high carbon concentrations taken during microfiltration and from the feed of nanofiltration (n = 5). BL = similar Blankaart water samples with low carbon concentration after nanofiltration treatment (n = 3). Absolute values are found in Table S4.

The recovery of TOC for real water samples was assessed by analysing Blankaart water at different stages in the drinking water treatment plant and comparing the total TOC concentration measured with HPSEC-TOC (with the Sievers^®^ M9) with the concentration obtained with the Shimadzu TOC V_CSH_ which is used as a reference. The recovery was 80 ± 10% (Table S3) and in line with the results for the model compounds. Next, the performance of the Sievers^®^ M9 in online mode and the performance of the Gräntzel thin-film reactor in online and offline mode were benchmarked against the total TOC concentration from the offline Sievers^®^ 900 as a reference for detectors using chemical oxidation. However, it should be noted that the recoveries obtained for the Sievers^®^ M9 compared to the Sievers^®^ 900 were similar as compared to the Shimadzu TOC V_CSH_ (Table S3 and Figure 9). For this, different Blankaart water samples were used which were obtained via membrane treatment.

The Gräntzel thin-film reactor obtained recoveries between 48–96% in offline mode (solely due to incomplete oxidation) and between 40–82% in online mode (combination of oxidation and adsorption) which are consistently lower than the recoveries from the Sievers^®^ M9 in online mode. Especially in samples with low carbon concentration (i.e., BL, Figure 9), the yield was ≤50%. This shows that a complete recovery of organic molecules is difficult to reach. Organic matter can adsorb to the column, but the oxidation efficiency during online measurements can also be impeded by the limited residence time of the sample in the detector. Since the same type of SEC column and operating conditions were used in online mode for the two types of detectors, it is assumed that the percentage of organic matter that adsorbed onto the column is similar. The different recoveries are therefore explained by the fundamental differences in both oxidation and detection between the two detectors. For oxidation, the Gräntzel thin-film reactor uses UV radiation where, in the Sievers^®^ M9, ammonium persulfate is dosed in addition to UV radiation which promotes the oxidation and therefore yields higher recoveries. However, the detection of CO_2_ through infra-red in the Gräntzel thin-film reactor is much more sensitive compared to the detection through conductivity in the Sievers^®^ M9, which probably caused the higher LOD and LOQ values of the HPSEC-TOC system in this work.

These observations are confirmed in the work of Lankes et al. (2009) where different organic carbon detectors are evaluated in their ability to oxidize NOM samples from aquatic environments [50]. The detector using high-temperature catalytic oxidation with infrared detection was assumed to yield a 100% oxidation efficiency of all compounds inside the sample [49,50]. This value was compared on the one hand with a UV-promoted wet-chemical oxidation using ammonium peroxodisulfate, which is similar to the oxidation used in this work, and on the other hand with a UV oxidation in a Gräntzel thin-film reactor. The Gräntzel thin-film reactor was only able to find between 70.9–93.0% of organic carbon compared to the catalytic oxidation, where it was between 85.3–105.2% for the wet-chemical oxidation, confirming the importance of persulfate addition to improve the oxidation [50]. This proves that the selected detector in our study is the most accurate one to quantify NOM in fresh water samples.

#### 2.2.3. Measurement Uncertainty

The results (in duplicate) from ten different real water samples in terms of average concentration of each defined MW fraction and their respective deviation (d) are presented in Table 2 (concentrations lower than the LOQ are not reported). Treated samples were measured after a membrane filtration. The relative measurement uncertainty U_rw_ for the high, medium and low MW NOM fractions are 3.86%, 3.22% and 5.17% respectively. These results show that the low MW integration range is the most challenging fraction in terms of measurement uncertainty. The low MW fraction is a tail rather than a peak, being very wide and not high which might explain the higher deviations found for this fraction. The obtained deviations are in line with the deviations reported for LC-OCD-OND analyses (see also Section 2.2.2c). Moreover, the concentration of the low MW fraction is relatively low in untreated surface water compared to e.g., the medium MW fraction. Its increased U_rw_ can be justified by the Horwitz ratio, which is a measure for the performance of an analytical method with respect to its trueness [51]. When applying the Horwitz equation on the concentration measured in e.g., sample D from Table 2, the acceptable coefficients of variation (CV) for the medium and low MW fraction are 17 and 22% respectively, which is far above the obtained values from our study. The selected samples for the description of the measurement uncertainty span a large variety of fresh water sources which were subjected to different treatment procedures. Since the overall relative measurement uncertainties do not exceed 4% for the high and medium MW fraction and is below 6% for the low MW fraction, it can be concluded that the HPSEC-TOC method is reliable and thus widely applicable for the analysis of fresh water samples having varying characteristics.

Next, Blankaart water was spiked with 2.5 mgC dm^−3^ isopropyl alcohol. This molecule was selected since it elutes in the low MW integration zone which is the most challenging. It is thus expected that the measurement uncertainty on the bias and the expanded measurement uncertainty obtained by spiking isopropyl alcohol in a real water sample will result in a poorer result compared to those resulting from spiking a molecule in the high or medium MW integration range. The measurement uncertainty on the bias U_bias_ was 8.73%, while the U_rw_ for the low MW fraction was 5.17%. The resulting overall expanded measurement uncertainty U was 20.30%, which is in line with the expectation based on the previous validation results in this work. These values should be taken into account when analysing unknown samples but fall within acceptable ranges as indicated in Table 2.

### 2.3. Sample Stability

**Figure 10:**
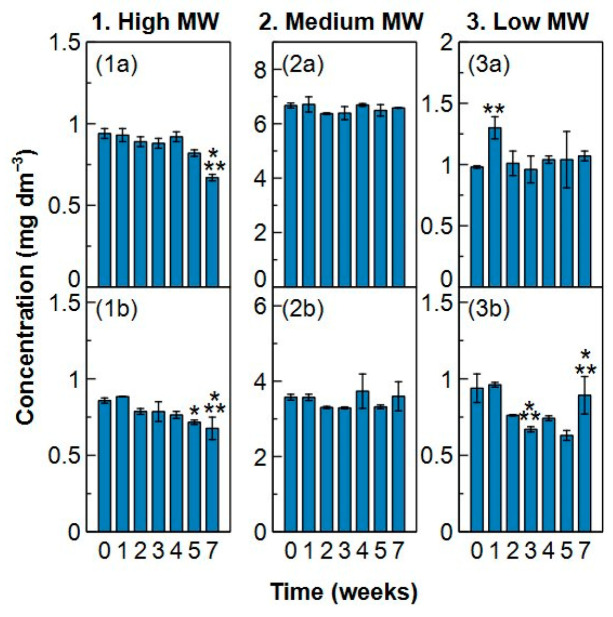
Concentration (mgC dm^−3^) in the high, medium and low molecular weight (MW) fractions of (**1a**–**3a**) Blankaart and (**1b**–**3b**) Coupure water in function of time (weeks) when preserved in the fridge at 5 °C. * = significantly different (*p* < 0.05) from week 1; ** = significantly different (*p* < 0.05) from week 0. Statistics were executed through a permutation test. Error bars show the standard deviation of two independent samples.

**Figure 11:**
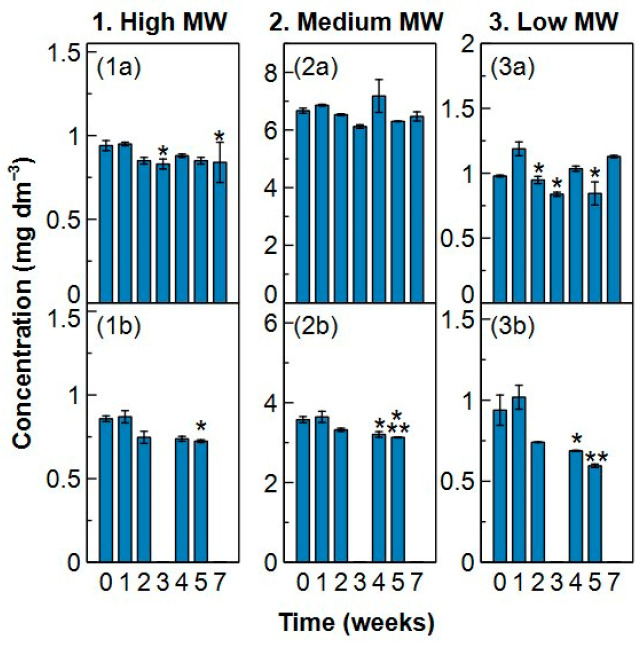
Concentration (mgC dm^−3^) in the high, medium and low molecular weight (MW) fractions of (**1a**–**3a**) Blankaart and (**1b**–**3b**) Coupure water in function of time (weeks) when preserved in the freezer at −18 °C. * = significantly different (*p* < 0.05) from week 1; ** = significantly different (*p* < 0.05) from week 0. Statistics were executed through a permutation test. Error bars show the standard deviation of two independent samples.

Coupure and Blankaart water were pre-filtered with a 6 µm filter and subsequently filtered with a 0.1 µm microfiltration membrane filter to remove the particulate organic matter without loss of dissolved organic carbon (Figure S8). Samples were kept in the fridge (5 °C) and freezer (−18 °C) for 7 weeks to find the best way to preserve fresh water samples. The change in concentration over time for each MW fraction can be found in Figure 10 and Figure 11.

The high MW fraction underwent no change in the fridge for at least 4 weeks for both types of surface water. In week 5 and week 7, a significant change in concentration occurred compared to week 0 and/or week 1. Although a small decrease in concentration in Coupure water (in the fridge) is seen from week 2, it only becomes significant from week 5 on. This decrease is also observed in the freezer samples for both waters. The decrease is only significant in week 3 and week 7 for Blankaart water and in week 5 for Coupure water. Therefore, when research interest goes to the high MW fraction, samples can be kept maximum 4 weeks in the fridge or 3 weeks in the freezer to maintain reliable results.

The medium MW fraction appears to stay stable in the fridge over the entire measurement period. However, Coupure water samples measured in week 4 and week 7 show more intra-variability (apparent from the high standard deviations). On the other hand, a significant decrease in concentration is observed from week 4 when Coupure water is kept in the freezer, while Blankaart water showed no changes. Since a higher variability was seen from week 4 onwards in samples with the same age, it is advised to not keep the samples for more than 4 weeks in the fridge nor the freezer for stable concentration measurements of the medium MW fraction in time.

The low MW fraction demonstrated significant variability between fridge samples measured in the same week (high standard deviations), as well as in different weeks. Blankaart water samples show one outlier in the first week and a high deviation between the measured samples in week 5, while the concentration in Coupure water decreased significantly in week 3 and showed high intra-variability in week 7. In the freezer samples, a significant decrease occurred in Blankaart water in week 2, 3 and 5 and from week 4 on in Coupure water. Consequently, analysis for this fraction should be done as soon as possible with a maximum delay of 2 weeks when the samples are stored in the fridge and a delay of 1 week when they are stored in the freezer. Low MW compounds are known to be easily consumed by micro-organisms, which might explain the instability of this fraction [52,53,54].

The TOC concentration (i.e., the sum of the fractions) changed significantly from the moment one of the individual fractions started to change, which in this case was the low MW fraction (Figure S9). The concentration in Blankaart samples from the fridge is significantly different in week 2 and week 3 compared to week 1, which is explained by the outlier seen for week 1 in the low MW fraction. The total concentration in Coupure samples from the fridge decreased from week 3, which is also the point where the concentration of the low MW fraction started decreasing. For the freezer samples, the decrease starts occurring from week 3 on for Blankaart water and from week 4 on for Coupure which is in agreement with the change seen in the low MW fraction. Therefore, analysis of TOC should be performed in the first 2 weeks after collection, both with fridge and freezer storage.

Furthermore, the pH of the samples remained more stable in the fridge than in the freezer (Figure S10). A very small, but significant decrease in pH is noted after three (Coupure) or four (Blankaart) weeks in the fridge. In the freezer, the samples tend to have a higher pH together with a very high variability in samples with the same storage time. Ion concentrations did not differ substantially from each other when stored in the fridge nor the freezer (Figures S11 and S12).

Overall, it is preferred to preserve fresh water samples in the fridge, since TOC concentrations and pH were more stable and less variable than for samples from the freezer. In week 7, turbidity was measured, because the freezer samples became more cloudy than the fridge samples. Indeed, the turbidity of Blankaart samples was almost 20 times higher in the freezer compared to the fridge (0.80 NTU vs. 15 NTU), indicating a change in water characteristics when freezing the samples. Significant and sometimes unpredictable changes in the optical properties or aromaticity of NOM by the process of freezing and thawing was also reported in literature [55,56,57,58,59]. Therefore, keeping samples in the fridge for two weeks seems the most appropriate method to ensure the stability of the sample. Sample storage in the fridge was also the preferred method in other studies investigating fresh water, tropical water or water from peatlands [55,56,57,58,59].

### 2.4. Application of the HPSEC-TOC Method in a Drinking Water Treatment Plant

**Figure 12:**
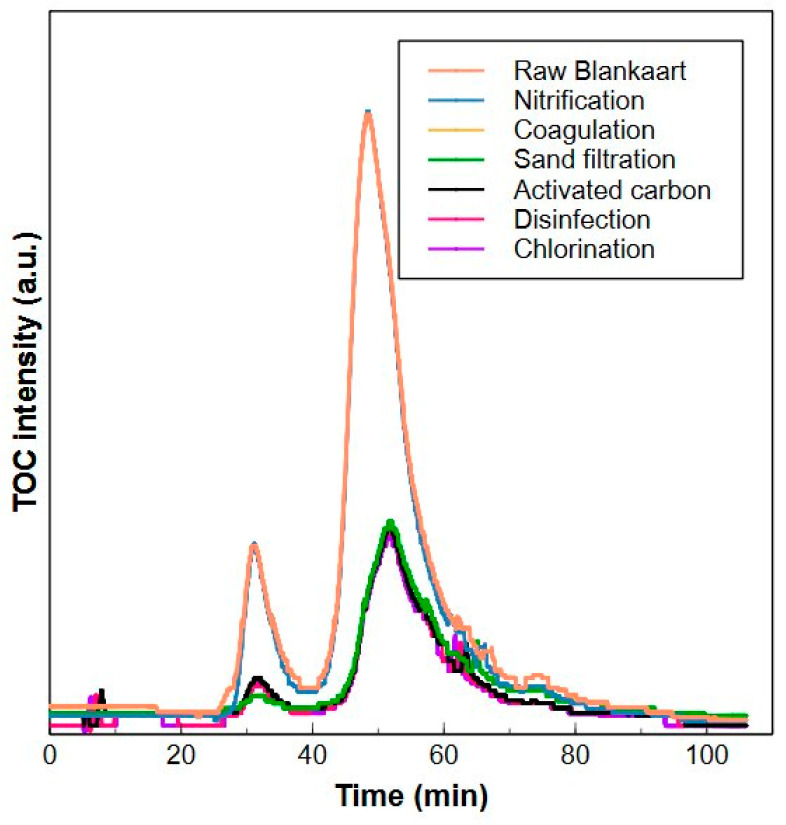
HPSEC-TOC chromatograms from every step in the drinking water treatment of Blankaart water. Raw Blankaart water is shown in orange, nitrification in blue, coagulation in yellow, sand filtration in green, activated carbon in black, disinfection in pink and the resulting drinking water after chlorination in purple.

The validated HPSEC method was put into practice to monitor the NOM removal in the drinking water treatment plant of De Blankaart. The treatment consists of a biological nitrification, coagulation, sand filtration, activated carbon and UV/chlorine disinfection (Figure 12). The sampling was executed in March 2023 and samples were measured within 2 weeks after sample collection.

The biological nitrification removed approximately 9.65% of the high MW fraction and 10.9% of the low MW fraction. No significant removal of the medium MW fraction was observed in this step. Coagulation was the most efficient, since it removed 70.3% of high MW fraction, 63.1% of the medium MW fraction and 7.20% of the low MW fraction. This is in accordance with other reported data using HPSEC-OCD having 32–50% biopolymer removal, 32–50% humic substances removal, 37–57% building blocks removal and 9–21% low MW neutral removal. The low MW acid fraction was hard to quantify due to the low concentration in the water [60,61]. The sand filter with intermediate chlorination could remove 55.2% of the remaining high MW fraction, 11.4% of the medium and 18.1% of the low MW fraction. Activated carbon was very efficient in removing the low MW fraction (33.4%) and part of the medium MW fraction (5.94%), which is in line with the findings of Gibert et al. (2013) [62], who investigated the removal of NOM fractions with HPSEC-OCD in a drinking water treatment plant using activated carbon and reported the highest removal efficiency for the low and intermediate MW NOM fractions. However, after the activated carbon, the concentration of the high MW fraction increased by 47.7%. This is a common phenomenon, since the biological activity within the activated carbon can excrete extracellular polymeric substances, causing a release of high MW molecules in the treated water [63,64]. Finally, UV/chlorine disinfection degraded 14.5% of the remaining high MW fraction, 3.6% of medium MW fraction and 8.8% of low MW fraction. This example shows the effectiveness of the validated HPSEC system to provide a fast and simple view on the treatment efficiency of the plant for the three fractions.

## 3. Materials and Methods

### 3.1. Chemicals

All solutions were prepared with ultrapure water from a MilliQ Millipore system. Sodium di-hydrogen phosphate (NaH_2_PO_4_, analytical grade) and potassium hydrogen carbonate (KHCO_3_, analytical grade) were purchased from VWR chemicals. Di-sodium hydrogen phosphate (Na_2_HPO_4_) and sodium sulfate (Na_2_SO_4_) were purchased from Supelco in analytical grade. 85% phosphoric acid (H_3_PO_4_) as well as sodium carbonate (Na_2_CO_3_) and potassium carbonate (K_2_CO_3_) were purchased from Merck. Analytical grade KHP powder was purchased from Acros organics, a certified reference material (50 mg dm^−3^ TOC KHP solution) was purchased from Chemlab. Pullulan and PEG were purchased from the Polymer Standards Service; sodium alginate and isopropylalcohol (HPLC grade) were obtained from Sigma-Aldrich and fumaric acid (for synthesis) from Merck.

The mobile phase was prepared by dissolving 2 mM NaH_2_PO_4_, 16 mM Na_2_HPO_4_ and 25 mM Na_2_SO_4_ in ultrapure water, the concentrated mobile phase with respectively 40 mM, 320 mM and 500 mM. Dilution series for KHP, Na_2_CO_3_ and K_2_CO_3_ were prepared from a stock solution of 200 mgC dm^−3^, 80 mgC dm^−3^ and 70 mgC dm^−3^ respectively in MilliQ. The 0.4 and 4.0 mgC dm^−3^ solution of the model compounds were prepared from a stock solution containing a concentration of 200 mgC dm^−3^ of each compound. Sodium alginate was first dissolved by ultrasonication, followed by dissolution of fumaric acid with ultrasonication. Isopropyl alcohol was added after stirring the solution overnight.

### 3.2. Water Sources

Several fresh water sources were used throughout the validation. The most frequently used water source was reservoir water from De Blankaart, a drinking water treatment plant from De Watergroep located in Diksmuide, Belgium. The reservoir is filled with water from the Ijzer river. The second water source, Coupure canal water located in Ghent, Belgium, was mostly used for IC removal tests (see Section 2.1.3) and for the sample stability tests (see Section 2.3). For the method measurement uncertainty (see Section 2.2.3), groundwater from Pidpa (located in Mol, Merksplas, Essen and Oud-Turnhout, Belgium), groundwater from Vitens (located in Spannenburg, The Netherlands) and tapwater from Farys (collected in Ghent, Belgium) were used. The main characteristics of the waters used is found in Table 3, detailed characteristics are found in Tables S1, S2 and S5–S7.

### 3.3. Instruments

#### 3.3.1. TOC Detectors

In this work, different TOC detectors (Table 4) were used for the validation. The detectors were operated either in online, *c.q.* they were coupled to an HPSEC-column, or offline mode, *c.q.* a bulk TOC measurement of the sample as such or via by-passing of the HPSEC-column. The Sievers^®^ M9 was used in the HPSEC-TOC configuration to measure the organic carbon concentration during SEC analysis, but also when the HPSEC column was by-passed. Here, organic matter is oxidized to CO_2_ by the addition of ammonium persulfate and subsequent UV irradiation (185 and 254 nm). This CO_2_ passes through a gas selective membrane, is dissolved on the other side of the membrane in demineralized water and subsequently measured through conductivity readings [44,65]. The Sievers^®^ 900 measures CO_2_ through the same principle, but was only used for offline measurements [65]. The Shimadzu TOC V_CPN_/V_CSH_ uses high temperature (680 °C) catalytic (platinum) oxidation for the conversion of organic matter into CO_2_ which is then measured with infrared detection [66]. This detector was used for IC analysis as well as offline TOC measurements. It is assumed that the high-temperature catalytic oxidation yields a 100% oxidation efficiency. Lastly, the Gräntzel thin-film reactor oxidizes organic carbon with a low-pressure mercury-vapor lamp and measures the produced CO_2_ with infrared. It was used in this work both in online and offline mode.

#### 3.3.2. HPSEC-TOC System

The analyses were performed using an Agilent 1260 HPLC system coupled with a TOC detector (Sievers^®^ M9 portable TOC analyser, Suez, Paris, France). A hydroxylated polymethylmethacrylate resin (TSK HW-50S, 20 mm × 250 mm, Tosoh Bioscience, South San Francisco, CA, USA) was used as stationary phase for the chromatographic separation. The mobile phase is a 4 mM phosphate buffer of pH 6.8 (2 mM NaH_2_PO_4_ and 1.6 mM Na_2_HPO_4_) with adapted ionic strength (25 mM Na_2_SO_4_) at a flowrate of 1 cm^3^ min^−1^. The choice for this mobile phase was motivated by the findings of Her et al. (2002). All samples were pretreated with a 20-fold concentrated mobile phase solution to assimilate the ionic environment of the samples (measured as conductivity) with that of the mobile phase to suppress matrix effects [20]. A constant level of conductivity of the samples is favorable for the organic carbon detection of the used system in this work, since the detection is based on conductometric measurements, even though the selective membrane is designed to theoretically only let the CO_2_-gas permeate. Samples were filtered with a 0.45 µm filter (Chromafil PET, Macherey-Nagel, Düren, Germany) before injection of 1350 mm^3^ in the system. This specific injection volume was used to optimize the sensitivity of the HPSEC-TOC system without overloading the SEC column [20]. The TOC analyser acidifies the influent (6 M phosphoric acid, 7 mm^3^ min^−1^) whereafter the IC, present as CO_2_ under the acidic conditions, is removed through a vacuum degasser. The organic carbon is converted into CO_2_ by a combination of UV radiation (185 and 254 nm) and ammonium persulfate addition as oxidizing reagent (15 *w*/*v*%, 4 mm^3^ min^−1^). In the measuring module of the TOC analyser, the formed CO_2_ passes through a selective membrane into the conductivity measuring cell [67]. In the turbo mode of the analyser, a datapoint is collected every 4 s. It must be noted that total exclusion of other ions such as Cl^−^ from the measuring module by the selective membrane cannot be guaranteed, making this detection technique less reliable for the measurement of samples with high conductivity such as marine waters. A universal interface box (UIB-II, Agilent, Santa Clara, CA, USA) was used to convert the current signal of the TOC analyser to a voltage signal, which is processed in the Agilent Open lab software (version 2.8). Three integration ranges were determined based on the analysis of surface water samples and will be referred to as the high, medium and low MW fraction of the sample. These ranges give both qualitative and quantitative information.

### 3.4. Sample Preparation

All samples measured in this work were subjected to the following procedure before HPSEC-TOC analysis:

1. The sample (aliquot of minimum 8 cm^3^ for practical reasons) was transferred into a TOC vial.
2. The pH (InoLab pH Level 1) of the sample was adjusted to pH 6 using 1 M H_3_PO_4_.
3. The sample was purged with N_2_-gas at 70 cm^3^ min^−1^ for at least 30 min.
4. A 20-times concentrated mobile phase solution was gradually added with a micropipette to the sample to assimilate the conductivity of the sample to the one of the mobile phase (5.3 mS).
5. A 0.45 µm filter (Chromafil PET-45/15, Macherey-Nagel) was pre-filtered with 2 mL sample to remove possible impurities of the filter whereafter another 2 mL was filtered and transferred into an HPLC vial.

### 3.5. Method Development

#### 3.5.1. Inorganic Carbon Removal

The Sievers^®^ M9 TOC detector is equipped with a tube degassing unit to remove IC before TOC measurements. However, in turbo mode, the capacity of this unit is insufficient to completely remove the IC from the samples during the HPSEC-TOC analysis, resulting in interference of the remaining IC with the TOC measurements. This was noticed by comparing the HPSEC-TOC chromatogram of a 40 mgC dm^−3^ KHCO_3_ sample prepared in MilliQ with a chromatogram of Blankaart water (Figure 3).

The efficiency of the degassing unit to remove IC was therefore investigated using IC standards. The standards ranged between 2.5 and 80 mgC dm^−3^ and were prepared using Na_2_CO_3_ (5, 15, 25, 40, 60, 80 mgC dm^−3^) or K_2_CO_3_ (2.5, 7.5, 10, 20, 30, 50, 70 mgC dm^−3^) in MilliQ. The standards were measured with the Shimadzu TOC V_CPN_ to determine the exact *IC* concentration of the prepared standards and with the HPSEC-TOC system to determine the amount of IC that was not removed by the degassing unit. The removal efficiency was thereafter calculated as

(1) Removal %=(ICShimadzu−ICHPSEC−TOC)ICShimadzu×100%

Next, Coupure water was adjusted to different pH values (5, 6, 7) with a 0.1 M H_3_PO_4_ solution. The water sample was either purged with N_2_-gas (Air Liquide, 99.999%; flowrate = 70 cm^3^ min^−1^) or sonicated in vacuum for 0, 10, 20, 30, 40, 50 or 60 min whereafter IC concentrations were measured (Shimadzu TOC V_CPN_) to determine which conditions can be used as sample pretreatment to completely remove the IC.

#### 3.5.2. Concentration and Molecular Weight Calibration of the HPSEC-TOC System

The TOC analyser was calibrated using KHP standards of 0.03, 0.04, 0.05, 0.06, 0.16, 0.30, 0.40, 0.80, 1.00, 1.20, 3.00, 4.00, 6.00, 8.00 and 10.00 mgC dm^−3^ in MilliQ. Pullulan and PEG standards were used for the MW calibration of the chromatographic system. Pullulan standards included 0.180, 0.342, 0.504, 1.03, 6.30, 9.80, 22.0 and 47.1 kDa, PEG standards included 0.238, 0.329, 0.599, 1.03, 1.53, 4.11, 3.45, 5.80, 11.4, 18.6, 25.3 and 44.0 kDa.

### 3.6. Method Validation

The method validation comprises the complete HPSEC-TOC method, including variations attributed by the instrument and by the manipulation or preparation of the water samples.

#### 3.6.1. Limit of Detection and Limit of Quantification

The LOD and LOQ of the method were determined by measuring a 250 µgC dm^−3^ KHP standard 10-fold. The LOD and LOQ were calculated as respectively 3 and 10 times the standard deviation on the mean value of the 10 measured concentrations [68].

#### 3.6.2. Precision and Trueness

- Certified reference material

A 2.5 mgC dm^−3^ KHP standard solution, prepared from a certified reference standard (50 mg dm^−3^ TOC) was measured six times over a period of 20 weeks in duplicate. The RSD and recovery was assessed. A non-parametric Kruskal-Wallis test was used followed by a Dunn’s multiple comparisons test (*p*-value = 0.05) to verify if the measurement was constant over time.

b. Model compounds

Sodium alginate, fumaric acid and isopropyl alcohol were spiked in MilliQ water at 0.4 mgC dm^−3^ and 4.0 mgC dm^−3^ for each compound to represent water samples having a low and rather high TOC content. The compounds were selected based on their difference in MW, each eluting in a particular MW fraction. The chemical structure and main properties of these three molecules are given in Table S8. Sodium alginate has the highest and isopropyl alcohol the lowest MW. Samples of both concentrations were divided into six individual vials, prepared and measured on the same day to determine the repeatability of the method in terms of RSD and recovery. By preparing and measuring three extra vials of each spiked concentration on a second day, the intra-repeatability was verified with a non-parametric Mann-Whitney U test to compare the two days (*p*-value = 0.05).

Adsorption of the compounds onto the HPSEC column was assessed only with the sample of high TOC content (4.0 mgC dm^−3^ spiked solution of each individual compound). The solution was first analysed with HPSEC-TOC and next with the Sievers^®^ M9 in offline mode, meaning that the column is by-passed. The difference in the concentration of each compound via both analysis is the part that is adsorbed onto the column.

c. Real water samples

Blankaart water was divided into six individual vials, prepared and measured to determine the repeatability of a real water sample in terms of RSD for each fraction. Furthermore, the total recovery of TOC with the HPSEC-TOC method was verified. For this, different samples of Blankaart water during drinking water treatment were taken and analysed with the Shimadzu TOC V_CSH_ and compared with the TOC concentration measured during a HPSEC-*TOC* analysis. The recovery was calculated as:

(2) Recovery %=TOCHPSEC−TOCTOCShimadzu

Next, a comparison was made between the Sievers^®^ M9 in online mode with the Gräntzel thin-film reactor both in online and offline mode. For this, samples were taken during a membrane treatment of Blankaart water. The treatment consisted of a filtration with a 6 µm filter (Whatmann filter paper, grade 3) followed by a 0.1 µm microfiltration (Synder Filtration MV0.1, flatsheet, Vacaville, CA, USA) and nanofiltration (Synder Filtration NFX, flatsheet) in a cross-flow filtration. Total TOC concentrations were measured with the Sievers^®^ 900 offline detector. It was assumed that this detector oxidized and thus recovered all organic carbon with chemical oxidation (see Section 2.2.2c). The recoveries in TOC concentration of these three systems were calculated based on the measurement with the Sievers^®^ 900.

#### 3.6.3. Method Measurement Uncertainty

The expanded measurement uncertainty of the method was determined by combining the in-lab reproducibility and the relative measurement uncertainty. First, ten different real water samples were analyzed, representing typical HPSEC-TOC routine analysis. The samples include Blankaart water (including microfiltered and nanofiltered samples), microfiltered Coupure water, groundwater from Pidpa (located in Mol, Merksplas, Essen and Oud-Turnhout, Belgium), groundwater from Vitens and tapwater from Farys.

The concentration for the high, medium and low MW fraction was determined in duplicate for each sample. The deviation (d) on the average value of the duplicate analysis was used in this case instead of the standard deviation which is typically used to express the variation of the average value for bigger data sets having a normal distribution:

(3) d %=|value1−value2|(value1 + value2)2×100%

The relative mean range (%R_mean_) was calculated as the average of d for the ten samples. The in-lab reproducibility of routine samples could be determined based on this data by calculating the RSD for each fraction, based on the relative differences of each fraction for every set of duplicates (*RSD_r_* = %Rmean1.128) [69]. An additional deviation factor (*RSD_rb_*, value has been set at 2.5%) was added to the *RSD_r_* to overcome differences related to analysis results gathered over a longer period of time and in this specific case to account for different types of water samples [69]. The overall relative measurement uncertainty on the in-lab HPSEC-TOC method is defined as *U_rw_*:

(4) $$U_{rw}\left(\%\right)=\sqrt{\left({RSD}_{r}^{2}+{RSD}_{rb}^{2}\right)}$$

This value is compared to the coefficient of variation (CV) determined by the Horwitz equation which is a measure for the performance of an analytical method with respect to its trueness [51]:

(5) $$CV=0.02\times {Concentration}^{-0.1505}$$

On the other hand, the relative measurement uncertainty on the bias (*U_bias_*) from recovery experiments is calculated by analyzing a real water sample spiked with 2.5 mgC dm^−3^ isopropyl alcohol (*IPA*) in 10-fold and determining the bias (*b_i_*) of each sample [69]:

(6) $$U_{bias}\;\left(\%\right)=\sqrt{\sum {(b}_{i}^{2}/10)}$$

(7) $${{With\;b}_{i}=[IPA]}_{measured,sample\;i}-{\left[IPA\right]}_{added}$$

The expanded measurement uncertainty (*U*) of the HSPEC-TOC method is calculated based on both the *U_rw_* and the *U_bias_* [69]:

(8) $$U\left(\%\right)=2\sqrt{\left(U_{rw}^{2}+U_{bias}^{2}\right)}$$

### 3.7. Sample Stability

The optimal conditions to preserve surface water samples for HPSEC-TOC analysis were determined by comparing two preservation conditions for two different surface waters, Blankaart and Coupure water respectively. Both waters were sampled on the same day, pre-filtered with a 6 µm filter (Whatmann filter paper, grade 3) and subsequently filtered with a 0.1 µm microfiltration membrane (Synder Filtration V0.1, cross-flow filtration) to remove the suspended and particulate organic matter. Filtration of the water within 24 h of sample collection impedes chemical and biological driven changes of the water [58]. The waters were stored in separate amber glass TOC vials in the fridge (5 °C) or freezer (−18 °C). The vials were pre-washed with 0.01 M HCl acid to prevent organic contamination [45]. HPSEC-TOC and pH analysis of each water were performed in duplicate on two different vials from both the fridge and freezer after 1, 2, 3, 4, 5 and 7 weeks. Ion analyses were conducted in week 1, 4 and 7 for both fridge and freezer samples in duplicate, using a Dionex Aquion Ion Chromatography System (ICP, Thermo Fisher, Waltham, MA, USA) equipped with a Dionex IonPac AS22 column for the anions and an ICP IRIS Intrepid II XSP (Thermo Fisher) system for the cations. Turbidity of the samples was measured in week 7 (Hanna Instruments, Washington, DC, USA, HI98703). All the results were compared through a permutation test which takes into account the exchangeability of the samples. The null hypothesis (*H_0_*) assumed for a certain parameter that no change occurred between two weeks, meaning:

(9) $$H_{0}:\;M_{x}=M_{y}$$

With *M_x_* the median for week x and *M_y_* the median for week y. The observed data from all weeks were randomly rearranged and the absolute difference in medians between two weeks was calculated for each rearrangement (100,000 iterations for HPSEC-TOC and pH results, 90 iterations for ion results). The *p*-value obtained from the permutation test represents the probability of obtaining the observed values assuming H_0_ is true. This *p*-value is compared to a significance level of 0.05. If the *p*-value is lower than this, H_0_ is rejected, meaning the two weeks under investigation differed significantly for a certain parameter. The permutation test was executed using Rstudio.

## 4. Conclusions

This work validated an optimized HPSEC-TOC method with the following outcome:

1. Both PEG and pullulan standards have been found suitable for the calibration of the system and as quality control for the separation performance of the SEC column.
2. Removal of IC by acidification of the sample to pH 6 (H_3_PO_4_) and subsequent purging prior to analysis avoids IC interference during the HPSEC-TOC method and does not modify the organic matter composition.
3. The LOD of the system is 19.0 µgC dm^−3^. The RSDs and recoveries for model compounds are respectively between 0.26–5.4% and 60–100%. For real water samples, the recovery was in general about 80%.
4. The relative measurement uncertainty U_rw_ on routine analysis of real water samples is between 3.22–5.17%, while the measurement uncertainty on the bias U_bias_, determined using a surface water sample spiked with isopropyl alcohol is 8.73%.
5. Analysis of a sample should be done after a maximum preservation of two weeks in the fridge to maintain the initial composition and characteristics of the water sample. Preservation in the freezer should be avoided.

The validated HPSEC-TOC method is an accessible, comprehensive and efficient tool for the characterization and quantification of NOM fractions in (treated) ground and surface water. The use of a single detector reduces the equipment cost and data-processing time extensively, while still providing a valuable addition to the more commonly used bulk techniques, such as UV and offline TOC. It allows an efficient monitoring of the water quality, and change thereof, in both research and industrial environments.

## Figures and Tables

**Table 1:** Recovery of sodium alginate, fumaric acid and isopropyl alcohol after passing through the HPSEC-TOC for 0.4 and 4.0 mgC dm^−3^ after two different days in 2022.

Recovery (%)	Sodium-Alginate		Fumaric Acid		Isopropyl Alcohol	
	19/09	21/09	19/09	21/09	19/09	21/09
0.4 mgC dm^−3^	59 ± 2	58 ± 4	70 ± 3	72 ± 4	90 ± 1	92 ± 1
4.0 mgC dm^−3^	67 ± 0.4	68 ± 0.8	94 ± 0.2	94 ± 1	100 ± 10	100 ± 1

**Table 2:** Average concentration of the high, medium and low MW fraction of routine duplicate analysis of 10 different (treated) real water samples, with their respective d-values and acceptable coefficient of variation (CV) according to the Horwitz equation.

Sample	Average Concentration (mgC dm^−3^)			d (%)			CV (%)		
	High MW	Medium MW	Low MW	High MW	Medium MW	Low MW	High MW	Medium MW	Low MW
A Blankaart 1	0.616	5.170	1.220	2.44	0.43	5.49	17	12	16
B Coupure microfiltrated	0.597	2.605	1.573	3.69	3.72	2.10	17	14	15
C Blankaart microfiltrated	0.554	4.407	1.109	6.50	0.41	9.56	17	13	16
D tap water	<LOQ ^a^	0.772	0.109	n.a.^b^	3.24	11.01	n.a. ^b^	17	22
E Essen groundwater	<LOQ ^a^	1.365	0.386	n.a. ^b^	0.22	10.12	n.a ^b^	15	18
F Spannenburg groundwater	<LOQ ^a^	6.543	0.723	n.a. ^b^	1.86	4.28	n.a ^b^	12	17
G Mol groundwater	<LOQ ^a^	0.524	0.365	n.a. ^b^	1.72	0.55	n.a. ^b^	18	19
H Merksplas groundwater	<LOQ ^a^	1.879	0.326	n.a. ^b^	1.97	4.29	n.a. ^b^	15	19
I Oud-Turnhout groundwater	<LOQ ^a^	2.122	0.443	n.a. ^b^	1.23	8.58	n.a. ^b^	14	18
J Blankaart nanofiltrated	0.314	0.090	2.056	0.64	6.67	0.63	19	23	14

^a^ Below limit of quantification (LOQ); ^b^ not applicable.

**Table 3:** Average parameters (pH, conductivity, ultraviolet absorption at 254 nm (UV_245_), total organic carbon (DOC) and inorganic carbon (IC)) of the water sources.

	pH (−)	Conduct. (µS cm^−1^)	UV_245_ (m^−1^)	TOC (mgC dm^−3^)	IC (mgC dm^−3^)
Blankaart	8.27 ± 0.03	800 ± 100	20 ± 3	7.7 ± 0.5	50 ± 10
Coupure	7.6 ± 0.7	800 ± 100	14 ± 4	8.5 ± 0.8	54 ± 5
Mol ^b^	8.1	219	1.7	0.8	21.77
Merksplas ^b^	7.7	430	7.2	2.7	50.33
Oud-Turnhout ^b^	7.7	314	7.1	2.9	37.01
Essen ^b^	8	344	5.9	2/0	40.74
Spannenburg	6.82 ± 0.08	660 ± 30	n.a. ^a^	n.a. ^a^	120 ± 10
Farys	7.8 ± 0.2	500 ± 100	n.a. ^a^	2 ± 1	40 ± 10

^a^ not available; ^b^ Pidpa only reported median values for these waters.

**Table 4:** Overview of the organic carbon detectors used in this work with their main characteristics.

	Oxidation	Detection	Used Mode ^a^
Sievers^®^ M9	photochemical + ammonium persulfate	Conductometric	Online/offline
Sievers^®^ 900	photochemical + ammonium persulfate	Conductometric	offline
Shimadzu TOC VCPN/VCSH	High-temperature catalytic combustion	Non dispersive infrared	offline
Gräntzel thin-film reactor	Photochemical	Infrared	Online/offline

^a^ Online = used in HPSEC-configuration, offline = bulk measurement/SEC column by-passed.

## Data Availability

The original contributions presented in the study are included in the article/Supplementary Material, further inquiries can be directed to the corresponding author/s.

## Supplementary Materials

The following supporting information can be downloaded at: https://www.mdpi.com/article/10.3390/molecules29092075/s1. Table S1. General water parameters from Blankaart water measured over the course of 2 years (2022–2023). Table S2. General water parameters from Coupure river water measured over the course of 2 years (2020–2021). Table S3. Analysis of Blankaart water during drinking water treatment from two different sampling rounds (March & October 2023) with catalytic oxidation (Shimadzu TOC V_CSH_) and an HPSEC-TOC analysis using Sievers^®^ M9 detector in online mode. The catalytic oxidation is assumed to reach 100% oxidation yield. The recovery of the HPSEC-TOC analysis is calculated based on the results of the catalytic oxidation. Standard deviations arise from technical replicates (n = 3). Table S4. Organic carbon concentration (mgC dm^−3^) of different reservoir samples during pre-filtration with a 6 µm and 0.1 µm filter followed by a nanofiltration. The samples were measured through different TOC detectors. Table S5. General ground water characteristics from Pidpa water measured in 2022 (reported values are median). Table S6. General water parameters from Spannenburg taken over the course of 3 years (2021–2023). Table S7. General tapwater characteristics from Farys water measured in 2022 in Ghent city. Table S8. Chemical structure and main properties of used model compounds. Figure S1. Calibration of the HPSEC-TOC system with potassium hydrogen phthalate standards between 0.03 and 10.0 mgC dm^−3^. Fit: y = 1617 x with R^2^ = 0.999. Figure S2. Chemical structure of pullulan (a), polyethylene glycol (b) and polystyrene sulfonate (c). Figure S3. HPSEC-TOC chromatogram of a reservoir water sample with indication of apparent molecular weight according to calibration on PEG and pullulan standards for the medium molecular weight fraction (see molecular weights indicated at 50 and 60 min). The difference in apparent molecular weight between the two calibrants does not exceed 10%, while for the high (40 min) and low (70 min) molecular weight fractions, the differences are more pronounced. Figure S4. Visualisation of the integration limit separating the medium MW zone from the low MW zone (vertical green line) on the chromatogram of a monovalent low molecular weight acid (fumaric acid). Figure S5. Removal (%) of inorganic carbon in function of inorganic carbon concentration (mgC dm^−3^) measured through HPSEC-TOC. The TOC detector removes between 94–98% of inorganic carbon. Figure S6. HPSEC-TOC chromatogram of Blankaart water, acidified to pH 6 (red), pH 4 (blue) and pH 2.5 (yellow) with subsequent N_2_-purging. Figure S7. Absolute loss of sodium alginate, fumaric acid and isopropyl alcohol standards after HPSEC-TOC analysis at a concentration of 0.4 mgC dm^−3^ (red solid bars) and 4.0mgC dm^−3^ (blue crossed bars). Figure S8. HPSEC-TOC chromatogram of raw (orange), buchner (6 µm; blue) and micro (0.1 µm; green) filtrated Blankaart water. Before analysis, all samples are filtered through a 0.45 µm filter to prevent the injection of any particles in the HPSEC-TOC system. Figure S9. Concentration (mgC dm^−3^) of total organic carbon in the fridge (5 °C) and the freezer (−18 °C) of (a) Blankaart and (b) Coupure in function of time (weeks). * = significantly different (*p* < 0.05) from week 1; ** = significantly different (*p* < 0.05) from week 0. Statistics were executed through a permutation test. Error bars show the standard deviation of two independent samples. Figure S10. pH of (a) Blankaart and (b) Coupure preserved in the fridge (5 °C) and the freezer (−18 °C) in function of time (weeks). ** = significantly different (*p* < 0.05) from week 0. Statistics were executed through a permutation test. Error bars show the standard deviation of two independent samples. Figure S11. Ion concentrations in Blankaart water at week 1,4 and 7 preserved in the fridge (5 °C, solid bar) and the freezer (−18 °C, crossed bar). (a) Fluoride, (b) Chloride, (c) Bromide, (d) Nitrate, (e) Phosphate, (f) Sulphate, (g) Calcium, (h) Potassium, (i) Sodium, (j) Magnesium. * = significantly different (*p* < 0.05) from week 1, ** = significantly different (*p* < 0.05) from week 0. Statistics were executed through a permutation test. Error bars show the standard deviation of two independent samples. Figure S12. Ion concentrations in Coupure water at week 1,4 and 7 preserved in the fridge (5 °C, solid bar) and the freezer (−18 °C, crossed bar). (a) Fluoride, (b) Chloride, (c) Bromide, (d) Nitrate, (e) Phosphate, (f) Sulphate, (g) Calcium, (h) Potassium, (i) Sodium, (j) Magnesium. * = significantly different (*p* < 0.05) from week 1, ** = significantly different (*p* < 0.05) from week 0. Statistics were executed through a permutation test. Error bars show the standard deviation of two independent samples.

## List of Abbreviations

(apparent) molecular weight: (a)MW, coefficient of variation: CV, deviation: d, expanded measurement uncertainty: U, Fourier-transform ion cyclotron resonance mass spectrometry: FTICR-MS, (high performance) size exclusion chromatography: (HP)SEC, inorganic carbon: IC, ion chromatography system: ICP, limit of detection: LOD, limit of quantification: LOQ, liquid chromatography: LC, measurement uncertainty on the bias: U_bias_, natural organic matter: NOM, nuclear magnetic resonance: NMR, organic carbon detector: OCD, organic nitrogen detector: OND, polyethylene glycol: PEG, potassium hydrogen phthalate: KHP, Pyrolysis-gas chromatography-mass spectrometry: Py-GC-MS, relative mean range: R_mean_, relative measurement uncertainty: U_rw_, relative standard deviation: RSD, total organic carbon: TOC, ultraviolet: UV.

## References

[1] Leenheer J.A., Croue J.P. (2003). Characterizing aquatic dissolved organic matter. Environ. Sci. Technol..

[2] Matilainen A., Gjessing E.T., Lahtinen T., Hed L., Bhatnagar A., Sillanpaa M. (2011). An overview of the methods used in the characterisation of natural organic matter (NOM) in relation to drinking water treatment. Chemosphere.

[3] Riyadh A., Peleato N.M. (2024). Natural Organic Matter Character in Drinking Water Distribution Systems: A Review of Impacts on Water Quality and Characterization Techniques. Water.

[4] Anderson L.E., DeMont I., Dunnington D.D., Bjorndahl P., Redden D.J., Brophy M.J., Gagnon G.A. (2023). A review of long-term change in surface water natural organic matter concentration in the northern hemisphere and the implications for drinking water treatment. Sci. Total Environ..

[5] Dejaeger K., Criquet J., Vanoppen M., Vignal C., Billon G., Cornelissen E.R. (2022). Identification of disinfection by-product precursors by natural organic matter fractionation: A review. Environ. Chem. Lett..

[6] Delpla I., Jung A.V., Baures E., Clement M., Thomas O. (2009). Impacts of climate change on surface water quality in relation to drinking water production. Environ. Int..

[7] Brezinski K., Gorczyca B. (2019). An overview of the uses of high performance size exclusion chromatography (HPSEC) in the characterization of natural organic matter (NOM) in potable water, and ion-exchange applications. Chemosphere.

[8] Zark M., Dittmar T. (2018). Universal molecular structures in natural dissolved organic matter. Nat. Commun..

[9] Ignatev A., Tuhkanen T. (2019). Step-by-step analysis of drinking water treatment trains using size-exclusion chromatography to fingerprint and track protein-like and humic/fulvic-like fractions of dissolved organic matter. Environ. Sci.-Water Res. Technol..

[10] Allpike B.P., Heitz A., Joll C.A., Kagi R.I., Abbt-Braun G., Frimmel F.H., Brinkmann T., Her N., Amy G. (2005). Size exclusion chromatography to characterize DOC removal in drinking water treatment. Environ. Sci. Technol..

[11] Wilske C., Herzsprung P., Lechtenfeld O.J., Kamjunke N., Einax J.W., von Tümpling W. (2021). New Insights into the Seasonal Variation of DOM Quality of a Humic-Rich Drinking-Water Reservoir-Coupling 2D-Fluorescence and FTICR MS Measurements. Water.

[12] Pan Y., Li H., Zhang X.R., Li A.M. (2016). Characterization of natural organic matter in drinking water: Sample preparation and analytical approaches. Trends Environ. Anal. Chem..

[13] Matilainen A., Sillanpaa M. (2010). Removal of natural organic matter from drinking water by advanced oxidation processes. Chemosphere.

[14] Xu J., Wang Y.Q., Gao D., Yan Z.R., Gao C., Wang L.L. (2017). Optical properties and spatial distribution of chromophoric dissolved organic matter (CDOM) in Poyang Lake, China. J. Great Lakes Res..

[15] Cai M.H., Wu Y.P., Ji W.X., Han Y.Z., Li Y., Wu J.C., Shuang C.-D., Korshin G.V., Li A.-M., Li W.-T. (2020). Characterizing property and treatability of dissolved effluent organic matter using size exclusion chromatography with an array of absorbance, fluorescence, organic nitrogen and organic carbon detectors. Chemosphere.

[16] Shimotori K., Satou T., Imai A., Kawasaki N., Komatsu K., Kohzu A., Tomioka N., Shinohara R., Miura S. (2016). Quantification and characterization of coastal dissolved organic matter by high-performance size exclusion chromatography with ultraviolet absorption, fluorescence, and total organic carbon analyses. Limnol. Oceanogr. Methods.

[17] Xu H.C., Guo L.D. (2017). Molecular size-dependent abundance and composition of dissolved organic matter in river, lake and sea waters. Water Res..

[18] Raeke J., Lechtenfeld O.J., Wagner M., Herzsprung P., Reemtsma T. (2016). Selectivity of solid phase extraction of fresh water dissolved organic matter and its effect on ultrahigh resolution mass spectra. Environ. Sci.-Process. Impacts.

[19] Stalter D., Peters L.I., O’Malley E., Tang J.Y.M., Revalor M., Farré M.J., Watson K., von Gunten U., Escher B.I. (2016). Sample Enrichment for Bioanalytical Assessment of Disinfected Drinking Water: Concentrating the Polar, the Volatiles, and the Unknowns. Environ. Sci. Technol..

[20] Her N., Amy G., Foss D., Cho J., Yoon Y., Kosenka P. (2002). Optimization of method for detecting and characterizing NOM by HPLC-size exclusion chromatography with UV and on-line DOC detection. Environ. Sci. Technol..

[21] Huber S.A., Balz A., Abert M., Pronk W. (2011). Characterisation of aquatic humic and non-humic matter with size-exclusion chromatography—Organic carbon detection—Organic nitrogen detection (LC-OCD-OND). Water Res..

[22] Mapp K. (2023). Size Exclusion Chromatography—Toyopearl Resins for SEC.

[23] Ruhl A.S., Jekel M. (2012). Elution behaviour of low molecular weight compounds in size exclusion chromatography. J. Water Supply Res. Technol.-AQUA.

[24] Hidayah E.N., Chou Y.C., Yeh H.H. (2016). Using HPSEC to identify NOM fraction removal and the correlation with disinfection by-product precursors. Water Sci. Technol.-Water Supply.

[25] Mojela H., Gericke G., Madhav H., Malinga S.P. (2023). Seasonal variations of natural organic matter (NOM) in surface water supplied to two coal-fired power stations. Environ. Sci. Pollut. Res..

[26] MacKeown H., Gyamfi J.A., Delaporte M., Schoutteten K., Verdickt L., Ouddane B., Criquet J. (2021). Removal of disinfection by-product precursors by ion exchange resins. J. Environ. Chem. Eng..

[27] Carra I., Lozano J.F., Johannesen S., Godart-Brown M., Goslan E.H., Jarvis P., Judd S. (2021). Sorptive removal of disinfection by-product precursors from UK lowland surface waters: Impact of molecular weight and bromide. Sci. Total Environ..

[28] Kaarela O., Koppanen M., Kesti T., Kettunen R., Palmroth M., Rintala J. (2021). Natural organic matter removal in a full-scale drinking water treatment plant using ClO_2_ oxidation: Performance of two virgin granular activated carbons. J. Water Process Eng..

[29] Lavanya G.S.M., Eswarudu M.M., Eswaraiah M.C., Harisudha K., Spandana B.N. (2013). Analytical Method Validation: An Updated Review. Int. J. Pharm. Sci. Res..

[30] Magnusson B., Örnemark U. (2014). Eurachem Guide: The Fitness for Purpose of Analytical Methods—A Laboratory Guide to Method validation and Related Topics.

[31] (2018). Water Quality—Sampling—Part 3: Preservation and Handling of Water Samples.

[32] Peters F.T., Drummer O.H., Musshoff F. (2007). Validation of new methods. Forensic Sci. Int..

[33] Dulaquais G., Breitenstein J., Waeles M., Marsac R., Riso R. (2018). Measuring dissolved organic matter in estuarine and marine waters: Size-exclusion chromatography with various detection methods. Environ. Chem..

[34] Park Y.J., Choi Y.B., Oh S.B., Moon J., Truong T.Q., Huynh P.K., Kim S.M. (2024). Development and application of a high-performance liquid chromatography diode-array detection (HPLC-DAD) method for the simultaneous quantification of phenolic compounds in the aerial part of *Glehnia littoralis*. Appl. Biol. Chem..

[35] Zhang T., Lu J.F., Ma J., Qiang Z.M. (2008). Fluorescence spectroscopic characterization of DOM fractions isolated from a filtered river water after ozonation and catalytic ozonation. Chemosphere.

[36] Thurman E. (1985). Organic Geochemistry of Natural Waters.

[37] Warton B., Heitz A., Allpike B., Kagi R. (2008). Size-exclusion chromatography with organic carbon detection using a mass spectrometer. J. Chromatogr. A.

[38] Park M., Lee D., Snyder S.A. (2021). Deconvolution of Size Exclusion Chromatograms: New Insights into the Molecular Weight Distribution of Dissolved Organic Matter in Ozone and Biological Activated Carbon. Acs Es&T Water.

[39] Humbert H., Gallard H., Suty H., Croue J.P. (2005). Performance of selected anion exchange resins for the treatment of a high DOC content surface water. Water Res..

[40] Tan Y.R., Kilduff J.E. (2007). Factors affecting selectivity during dissolved organic matter removal by anion-exchange resins. Water Res..

[41] Sleighter R.L., Hatcher P.G. (2008). Molecular characterization of dissolved organic matter (DOM) along a river to ocean transect of the lower Chesapeake Bay by ultrahigh resolution electrospray ionization Fourier transform ion cyclotron resonance mass spectrometry. Mar. Chem..

[42] (2017). Choosing the Right Calibration for the Agilent Bio SEC-3 Column.

[43] Matilainen A., Vepsalainen M., Sillanpaa M. (2010). Natural organic matter removal by coagulation during drinking water treatment: A review. Adv. Colloid Interface Sci..

[44] (2014). Sievers M9/M9e TOC Analyzers—Operation and Maintenance Manual.

[45] Karanfil T., Schlautman M.A., Erdogan I. (2002). Survey of DOC and UV measurement practices with implications for SUVA determination. J. Am. Water Works Assoc..

[46] Dasaard C.B., Bayless D.J., Stuart B.J. (2016). Saturated pH and Total Inorganic Carbon from CO2 Solubility Related to Algal Growth. Int. Adv. Res. J. Sci. Eng. Technol..

[47] Allpike B.P., Heitz A., Joll C.A., Kagi R.I. (2007). A new organic carbon detector for size exclusion chromatography. J. Chromatogr. A.

[48] Zhang W.J., Li L., Wang D.H., Wang R., Yu S.L., Gao N.Y. (2022). Characterizing dissolved organic matter in aquatic environments by size exclusion chromatography coupled with multiple detectors. Anal. Chim. Acta.

[49] Li X., Rao N.R.H., Linge K.L., Joll C.A., Khan S., Henderson R.K. (2019). An evaluation of measurement techniques for algal-derived organic nitrogen. Water Res..

[50] Lankes U., Mueller M.B., Weber M., Frimmel F.H. (2009). Reconsidering the quantitative analysis of organic carbon concentrations in size exclusion chromatography. Water Res..

[51] Horwitz W., Albert R. (2006). The Horwitz ratio (HorRat): A useful index of method performance with respect to precision. J. AOAC Int..

[52] Hammes F., Meylan S., Salhi E., Koster O., Egli T., Von Gunten U. (2007). Formation of assimilable organic carbon (AOC) and specific natural organic matter (NOM) fractions during ozonation of phytoplankton. Water Res..

[53] Hem L.J., Efraimsen H. (2001). Assimilable organic carbon in molecular weight fractions of natural organic matter. Water Res..

[54] Vanderkooij D., Visser A., Hijnen W.A.M. (1982). Determining the concentration of easily assimilable organic-carbon in drinking-water. J. Am. Water Work Assoc..

[55] Payandi-Rolland D., Shirokova L.S., Labonne F., Benezeth P., Pokrovsky O.S. (2021). Impact of freeze-thaw cycles on organic carbon and metals in waters of permafrost peatlands. Chemosphere.

[56] Peacock M., Freeman C., Gauci V., Lebron I., Evans C.D. (2015). Investigations of freezing and cold storage for the analysis of peatland dissolved organic carbon (DOC) and absorbance properties. Environ. Sci.-Process. Impacts.

[57] Chen J., Xue S., Lin Y.Z., Wang C., Wang Q., Han Q. (2016). Effect of freezing-thawing on dissolved organic matter in water. Desalination Water Treat..

[58] Lloyd C.E.M., Johnes P.J., Pemberton J.A., Yates C.A., Jones D., Evershed R.P. (2022). Sampling, storage and laboratory approaches for dissolved organic matter characterisation in fresh waters: Moving from nutrient fraction to molecular-scale characterisation. Sci. Total Environ..

[59] Cook S., Peacock M., Evans C.D., Page S.E., Whelan M., Gauci V., Khoon K.L. (2016). Cold storage as a method for the long-term preservation of tropical dissolved organic carbon (DOC). Mires Peat.

[60] Baghoth S.A., Sharma S.K., Amy G.L. (2011). Tracking natural organic matter (NOM) in a drinking water treatment plant using fluorescence excitation-emission matrices and PARAFAC. Water Res..

[61] Krzeminski P., Vogelsang C., Meyn T., Köhler S.J., Poutanen H., de Wit H.A., Uhl W. (2019). Natural organic matter fractions and their removal in full-scale drinking water treatment under cold climate conditions in Nordic capitals. J. Environ. Manag..

[62] Gibert O., Lefèvre B., Fernández M., Bernat X., Paraira M., Pons M. (2013). Fractionation and removal of dissolved organic carbon in a full-scale granular activated carbon filter used for drinking water production. Water Res..

[63] Bi Z.H., Li T., Xing X.C., Qi P., Li Z.S., Hu C., Xu X., Sun Z., Xu G., Chen C. (2022). Contribution of extracellular polymeric substances and microbial community on the safety of drinking water quality: By mean of Cu/activated carbon biofiltration. Chemosphere.

[64] Shen H., Tang X.C., Wu N.X., Chen H.B. (2018). Leakage of soluble microbial products from biological activated carbon filtration in drinking water treatment plants and its influence on health risks. Chemosphere.

[65] Godec R., O’Neill K.J., Hutte R. (1999). Method and Apparatus for The Measurement of Dissolved Carbon.

[66] Ming C.A. (2003). High Sensitivity Total Organic Carbon Analysis.

[67] Godec R., Kosenka P., Hutte R. (1992). Method and Apparatus for the Determination of Dissolved Carbon in Water.

[68] Vial J., Jardy A. (1999). Experimental comparison of the different approaches to estimate LOD and LOQ of an HPLC method. Anal. Chem..

[69] FASFC (2009). Microbiology—Estimationg Measurement Uncertainty.

