# Supplementary material for: Thorough Validation of Optimized Size Exclusion Chromatography-Total Organic Carbon Analysis for Natural Organic Matter in Fresh Waters
Source: Molecules. 2024 Apr 30;29(9):2075. doi: 10.3390/molecules29092075 (PMC11085112; doi:10.3390/molecules29092075)
Supplement: Supplementary file 1 [file molecules-29-02075-s001.zip › molecules-2927927-supplementary.pdf]

Table S1. General water parameters from Blankaart water measured over the course of 2 years (2022-2023).

**pH (-)**	8.27 ± 0.03	**Chloride (mg dm^{-3})**	120 ± 40
**Conductivity (µS cm^{-1})**	800 ± 100	**Sulphate (mg dm^{-3})**	108 ± 5
**UV at 254nm (m^{-1})**	20 ± 3	**Sodium (mg dm^{-3})**	100 ± 50
**SUVA at 254nm (dm^{-3} mg^{-1} m^{-1})**	2.62 ± 0.08	**Potassium (mg dm^{-3})**	40 ± 20
**TOC (mgC dm^{-3})**	7.7 ± 0.5	**Calcium (mg dm^{-3})**	80 ± 30
**Inorganic carbon (mgC dm^{-3})**	50 ± 10	**Magnesium (mg dm^{-3})**	20 ± 10

Table S2. General water parameters from Coupure river water measured over the course of 2 years (2020-2021).

**pH (-)**	7.6 ± 0.7	**Chloride (mg dm^{-3})**	95 ± 6
**Conductivity (µS cm^{-1})**	800 ± 100	**Sulphate (mg dm^{-3})**	80 ± 10
**UV at 254nm (m^{-1})**	14 ± 4	**Sodium (mg dm^{-3})**	66 ± 4
**SUVA at 254nm (dm^{-3} mg^{-1} m^{-1})**	2.0 ± 0.3	**Potassium (mg dm^{-3})**	16.2 ± 0.8
**TOC (mgC dm^{-3})**	8.5 ± 0.8	**Calcium (mg dm^{-3})**	110 ± 20
**Inorganic carbon (mgC dm^{-3})**	55 ± 5	**Magnesium (mg dm^{-3})**	11 ± 2

Table S3. Analysis of Blankaart water during drinking water treatment from two different sampling rounds (March & October 2023) with catalytic oxidation (Shimadzu TOC V_{CSH}) and an HPSEC-TOC analysis using Sievers® M9 detector in online mode. The catalytic oxidation is assumed to reach 100% oxidation yield. The recovery of the HPSEC-TOC analysis is calculated based on the results of the catalytic oxidation. Standard deviations arise from technical replicates (n = 3).

	**Shimadzu TOC V_{CSH} (mgC dm^{-3})**		**Sievers M9 online recovery (%)**	
Sampling date	***27/04***	***04/10***	***27/04***	***04/10***
Sample 1	8.64 ± 0.01	10.4 ± 0.2	95.6 ± 0.1	108 ± 2
Sample 2	7.9 ± 0.2	10.9 ± 0.7	108 ± 3	107 ± 7
Sample 3	3.5 ± 0.2	5.3 ± 0.5	81 ± 4	91 ± 8
Sample 4	3.32 ± 0.02	5.6 ± 0.3	89.2 ± 0.5	78 ± 4
Sample 5	2.8 ± 0.1	3.9 ± 0.5	70 ± 3	90 ± 10
Sample 6	2.72 ± 0.09	3.9 ± 0.4	72 ± 3	90 ± 10
Sample 7	2.9 ± 0.1	3.6 ± 0.4	76 ± 3	100 ± 10

Table S4. Organic carbon concentration (mgC dm^{-3}) of different reservoir samples during pre-filtration with a 6µm and 0.1µm filter followed by a nanofiltration. The samples were measured through different TOC detectors.

	**Sievers 900 offline**	**Sievers M9 online**	**Gräntzel thin-film online**	**Gräntzel thin-film offline**
BH1 [a]	7.01	6.31	5.11	6.00
BH2 [a]	6.99	6.24	5.03	6.04
BH3 [a]	7.15	6.91	5.54	6.64
BH4 [a]	7.36	7.15	6.06	7.04
BL1 [b]	3.15	2.65	1.26	1.59
BL2 [b]	2.17	1.54	0.89	1.05
BH5 [a]	8.42	7.63	6.30	7.71
BL3 [b]	1.56	1.51	0.69	0.92

[a]Blankaart with high carbon content, [b]Blankaart with low carbon content

Table S5. General ground water characteristics from Pidpa water measured in 2022 (reported values are median).

	Mol	Merksplas	Oud-Turnhout	Essen
**pH (-)**	8.1	7.7	7.7	8
**Conductivity (µS cm^{-1})**	219	430	314	344
**UV at 254nm (m^{-1})**	1.7	7.2	7.1	5.9
**SUVA at 254nm (dm^{-3} mg^{-1} m^{-1})**	2.13	2.67	2.45	2.95
**TOC (mgC dm^{-3})**	0.8	2.7	2.9	2.0
**Inorganic carbon (mgC dm^{-3})**	21.77	50.33	37.01	40.74
**Chloride (mg dm^{-3})**	15.1	21.1	13.2	13.5
**Sulphate (mg dm^{-3})**	11.6	17.9	11.5	11.6
**Sodium (mg dm^{-3})**	26.3	11.6	7.5	11.1
**Potassium (mg dm^{-3})**	3.3	8.1	4.9	6.6
**Calcium (mg dm^{-3})**	20.0	72.6	48.2	57.5
**Magnesium (mg dm^{-3})**	2.8	6.7	8.3	4.3

Table S6. General water parameters from Spannenburg taken over the course of 3 years (2021-2023).

**pH (-)**	6.82 ± 0.08	**Chloride (mg dm^{-3})**	28 ± 8
**Conductivity (µS cm^{-1})**	650 ± 30	**Sulphate (mg dm^{-3})**	<2
**UV at 254nm (m^{-1})**	n.a.[a]	**Sodium (mg dm^{-3})**	17 ± 2
**SUVA at 254nm (dm^{-3} mg^{-1} m^{-1})**	n.a. [a]	**Potassium (mg dm^{-3})**	2.0 ± 0.2
**TOC (mgC dm^{-3})**	n.a. [a]	**Calcium (mg dm^{-3})**	120 ± 9
**Inorganic carbon (mgC dm^{-3})**	120 ± 10	**Magnesium (mg dm^{-3})**	11.36

[a]Not available

Table S7. General tapwater characteristics from Farys water measured in 2022 in Ghent city.

**pH (-)**	7.8 ± 0.2	**Chloride (mg dm^{-3})**	60 ± 30
**Conductivity (µS cm^{-1})**	500 ± 100	**Sulphate (mg dm^{-3})**	70 ± 20
**UV at 254nm (m^{-1})**	n.a. [a]	**Sodium (mg dm^{-3})**	40 ± 10
**SUVA at 254nm (dm^{-3} mg^{-1} m^{-1})**	n.a. [a]	**Potassium (mg dm^{-3})**	6 ± 3
**TOC (mg dm^{-3})**	2 ± 1	**Calcium (mg dm^{-3})**	80 ± 30
**Inorganic carbon (mg HCO_3 dm^{-3})**	200 ± 50	**Magnesium (mg dm^{-3})**	9.9

[a]Not available

Table S8. Chemical structure and main properties of used model compounds.

**Model compound**	**Chemical structure or building blocks**	**pK$_{a,1}$**	**Molecular weight (g mol^{-1})**	**Elution range HPSEC-TOC method**
Alginate	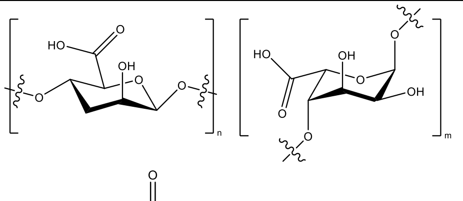	3.2	n.a.[a]	High MW
Fumaric acid	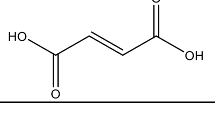	3.0	116.1	Medium MW

Iso-propyl alcohol	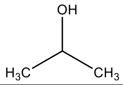	17.1	60.1	Low MW

[a]not available

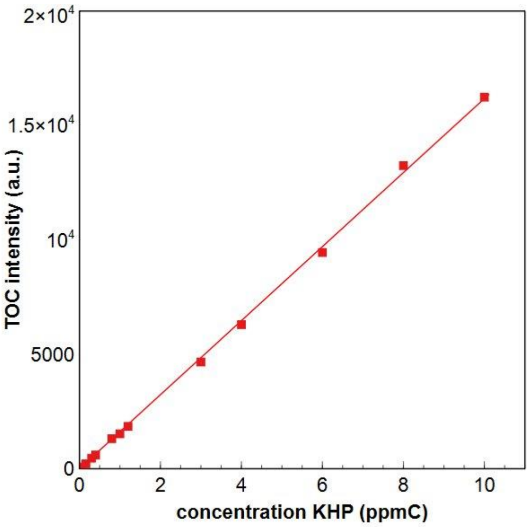

Figure S1. Calibration of the HPSEC-TOC system with potassium hydrogen phthalate standards between 0.03 and 10.0mgC dm^{-3}. Fit: y=1617x with R^2 = 0.999.

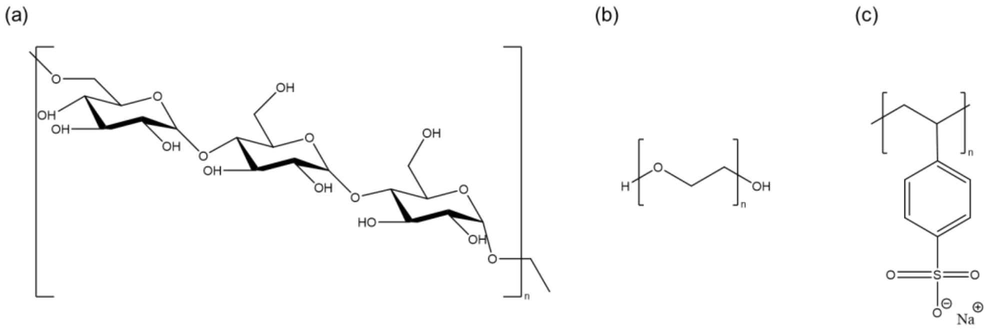

Figure S2. Chemical structure of pullulan (a), polyethylene glycol (b) and polystyrene sulfonate (c).

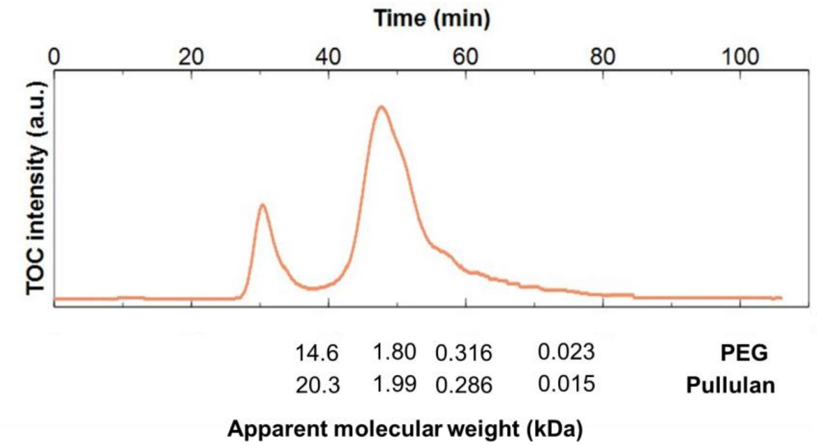

Figure S3. HPSEC- TOC chromatogram of a reservoir water sample with indication of apparent molecular weight according to calibration on PEG and pullulan standards for the medium molecular weight fraction (see molecular weights indicated at 50 and 60min). The difference in apparent molecular weight between the two calibrants does not exceed 10%, while for the high (40min) and low (70min) molecular weight fractions, the differences are more pronounced.

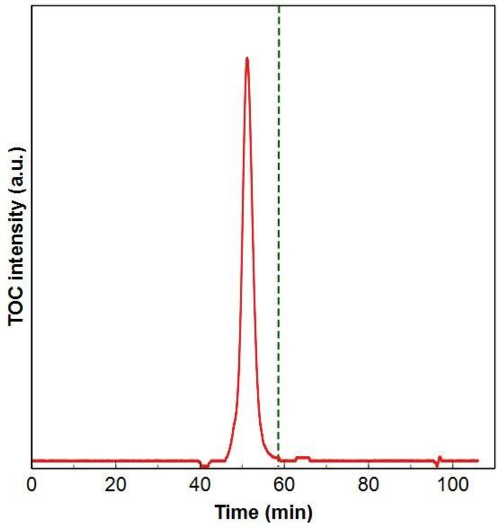

Figure S4. Visualisation of the integration limit separating the medium MW zone from the low MW zone (vertical green line) on the chromatogram of a monovalent low molecular weight acid (fumaric acid).

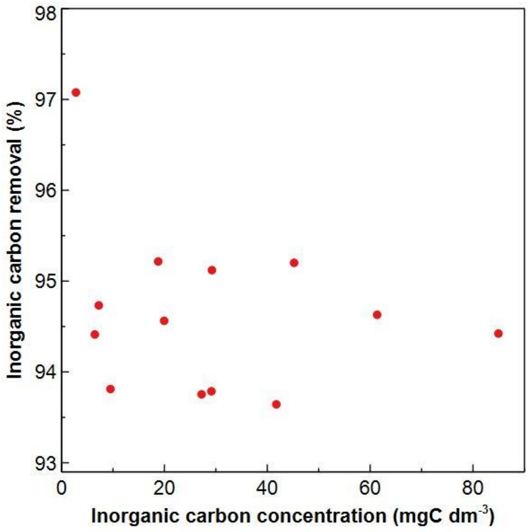

Figure S5. Removal (%) of inorganic carbon in function of inorganic carbon concentration (mgC dm^{-3}) measured through HPSEC-TOC. The TOC detector removes between 94 – 98% of inorganic carbon.

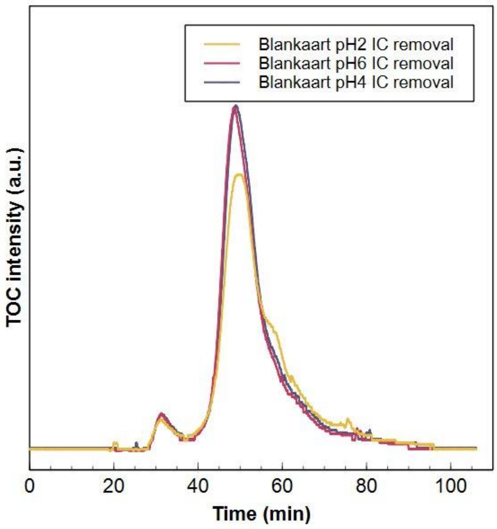

Figure S6. HPSEC-TOC chromatogram of Blankaart water, acidified to pH 6 (red), pH 4 (blue) and pH 2.5 (yellow) with subsequent N_2-purging.

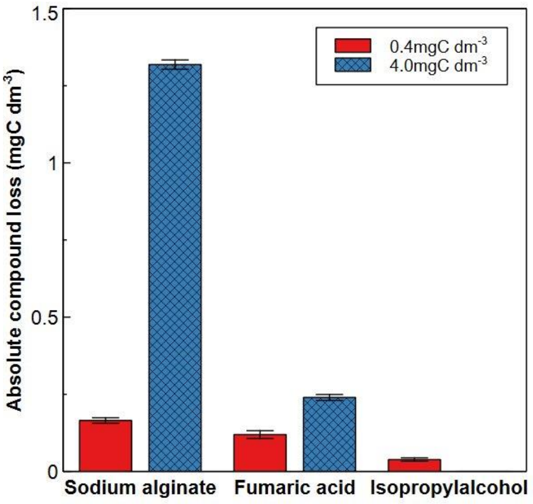

Figure S7. Absolute loss of sodium alginate, fumaric acid and isopropyl alcohol standards after HPSEC-TOC analysis at a concentration of 0.4mgC dm^{-3} (red solid bars) and 4.0mgC dm^{-3} (blue crossed bars).

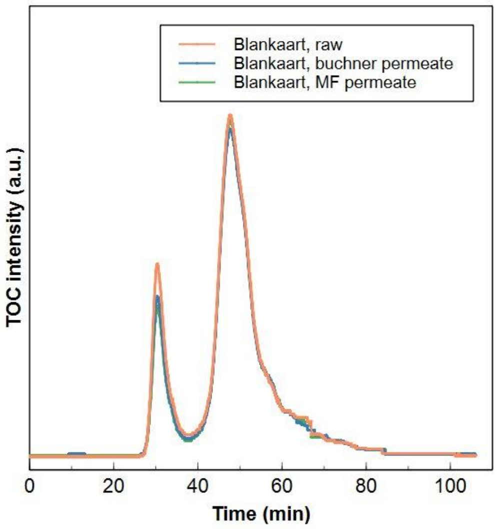

Figure S8. HPSEC-TOC chromatogram of raw (orange), buchner (6μm; blue) and micro (0.1μm; green) filtrated Blankaart water. Before analysis, all samples are filtered through a 0.45μm filter to prevent the injection of any particles in the HPSEC-TOC system.

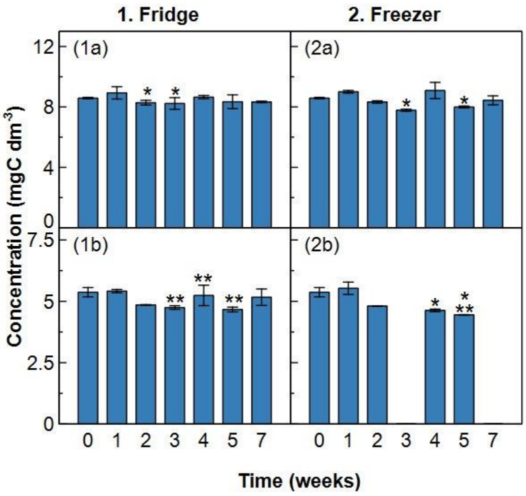

Figure S9. Concentration (mgC dm^{-3}) of total organic carbon in the fridge (5°C) and the freezer (-18°C) of (a) Blankaart and (b) Coupure in function of time (weeks). * = significantly different ($p < 0.05$) from week 1; ** = significantly different ($p < 0.05$) from week 0. Statistics were executed through a permutation test. Error bars show the standard deviation of two independent samples.

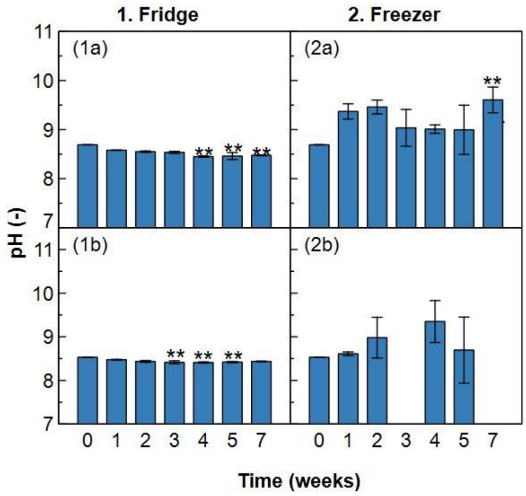

Figure S10. pH of (a) Blankaart and (b) Coupure preserved in the fridge (5°C) and the freezer (-18°C) in function of time (weeks). ** = significantly different ($p < 0.05$) from week 0. Statistics were executed through a permutation test. Error bars show the standard deviation of two independent samples.

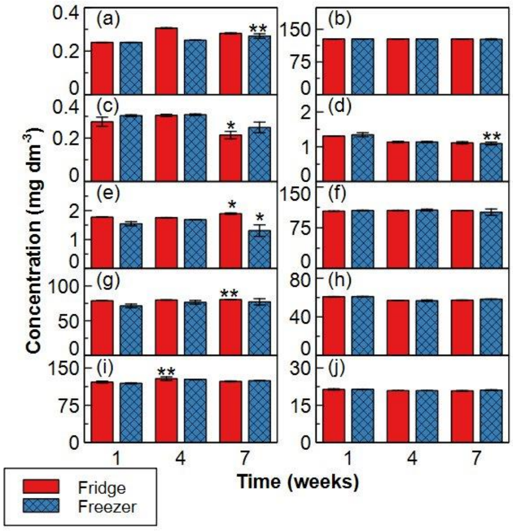

Figure S11. Ion concentrations in Blankaart water at week 1,4 and 7 preserved in the fridge (5°C, solid bar) and the freezer (-18°C, crossed bar). (a) Fluoride, (b) Chloride, (c) Bromide, (d) Nitrate, (e) Phosphate, (f) Sulphate, (g) Calcium, (h) Potassium, (i) Sodium, (j) Magnesium. * = significantly different ($p < 0.05$) from week 1, ** = significantly different ($p < 0.05$) from week 0. Statistics were executed through a permutation test. Error bars show the standard deviation of two independent samples.

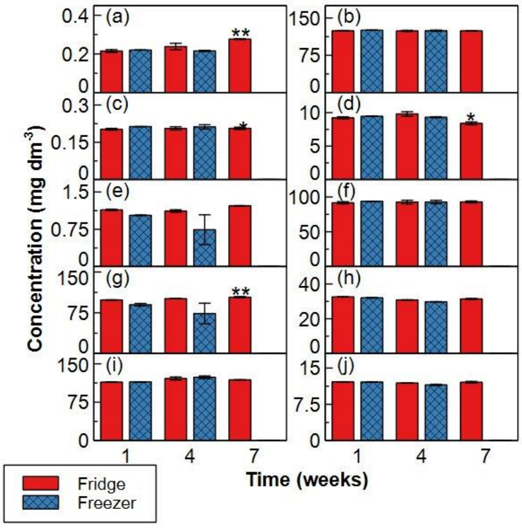

Figure S12. Ion concentrations in Coupure water at week 1,4 and 7 preserved in the fridge (5°C, solid bar) and the freezer (-18°C, crossed bar). (a) Fluoride, (b) Chloride, (c) Bromide, (d) Nitrate, (e) Phosphate, (f) Sulphate, (g) Calcium, (h) Potassium, (i) Sodium, (j) Magnesium. * = significantly different ($p < 0.05$) from week 1, ** = significantly different ($p < 0.05$) from week 0. Statistics were executed through a permutation test. Error bars show the standard deviation of two independent samples.
